# Sequence Diversity and Encoded Enzymatic Differences of Monocistronic L1 ORF2 mRNA Variants in the Aged Normal and Alzheimer's Disease Brain

**DOI:** 10.1523/JNEUROSCI.2298-24.2025

**Published:** 2025-05-14

**Authors:** Juliet Nicodemus, Christine S. Liu, Linnea Ransom, Valerie Tan, William Romanow, Natalia Jimenez, Jerold Chun

**Affiliations:** ^1^Sanford Burnham Prebys Medical Discovery Institute, La Jolla, California 92037; ^2^School of Medicine, University of California San Diego, La Jolla, California 92037

**Keywords:** genomic mosaicism, LINE1, neurodegeneration, retrotransposons, reverse transcriptase, somatic genomic mosaicism

## Abstract

Reverse transcriptase (RT) activity in the human brain has been inferred through somatic retroinsertion/retrotransposition events; however, actual endogenous enzymatic activities and sources remain unclear. L1 (LINE-1) retrotransposons bicistronically express ORF2, containing RT and endonuclease (EN) domains, and RNA-binding protein ORF1, together enabling L1 retrotransposition and contributing to somatic genomic mosaicism. Here, we assessed endogenous RT activities and L1 mRNA diversity from cerebral cortex samples of 31 Alzheimer's disease (AD) and nondiseased (ND) brains (both sexes) using enzymatic functional assays, targeted PacBio HiFi long-read sequencing, and quantitative spatial transcriptomics. Expected bicistronic, full-length L1 transcripts were absent from most samples, constituting <0.01% of L1 sequences, of which >80% were noncoding. Monocistronic ORF1 and ORF2 transcripts were identified across all samples, consistent with quantitative spatial transcriptomics that identified discordant ORF2 and ORF1 expression in neurons. All brains had RT activity, with AD samples showing less activity, consistent with neuronal loss of terminal AD versus aged ND donors. Brain RT activity was higher in the gray matter and correlated with increased neuronal ORF2 expression, further supporting neuronal contributions. Remarkably, >550 protein-encoding, poly(A^+^) ORF2 sequence variants were identified, over two times more than identified in the human reference genome (hg38). Experimental overexpression of full-length and truncated ORF2 variants revealed ∼50-fold RT and ∼1.3-fold EN activity ranges, supporting endogenous functional capacity of monocistronic ORF2 variants in the human brain. The vast sequence diversity of monocistronic ORF2 mRNAs could underlie functional differences in RT-mediated somatic gene recombination/retroinsertion and resulting genomic mosaicism in the normal and diseased brain.

## Significance Statement

Human brain reverse transcriptase (RT) activity has been inferred through the “copy-and-paste life-cycle” of L1, which can generate genomic mosaicism via self-retrotransposition via a full-length L1 mRNA. However, their presence in aged and Alzheimer's disease (AD) neurons remains unclear. We examined aged normal and Alzheimer's brains for RT activity in prefrontal and medial–temporal cortices and its relationship to L1 via enzymatic activity assays and targeted PacBio sequencing. RT activity was pervasive; however, full-length L1 was largely absent. Instead, hundreds of different, truncated, novel L1 mRNA variants were identified, and experimental sampling revealed diverse RT activities. These data implicate truncated L1 variants as a source of functionally diverse and novel RTs in the normal and AD brain.

## Introduction

The human brain is composed of a wide array of cell types intricately connected into networks that form the basis of neural function, especially through postmitotic neurons (Anda et al., 2016). Myriad DNA sequence changes have been documented in single neurons, constituting somatic genomic mosaicism (SGM; Richardson et al., 2014; Rohrback et al., 2018; Costantino et al., 2021). SGM more commonly affects noncoding sequences that represent over 98% of the human genome, but an exception that alters coding sequences has been reported (Lander et al., 2001; Lee et al., 2018, 2020; Park et al., 2019; Mitsunaga et al., 2023). Somatic gene recombination (SGR) involves the generation of intronless and nonannotated coding “genomic cDNAs” (gencDNAs) within genomic DNA of the brain, which has been associated with Alzheimer's disease (AD; Lee et al., 2018, 2020; Park et al., 2019; Mitsunaga et al., 2023). GencDNAs have coding potential, are inserted in novel genomic locations, can occur in many different forms for a single gene within one tissue and individual, and require an active reverse transcriptase (RT) in aging, postmitotic neurons (Lee et al., 2018, 2020; Park et al., 2019; Mitsunaga et al., 2023). However, little is known about actual endogenous enzymatic RT activity, cellular sources, or underlying expressed RT genes.

L1 (long interspersed element-1) retrotransposons are a likely source of RT activity through open reading frame 2 (ORF2) that can encode an active RT. The human genome contains ∼500,000 copies of L1 composing ∼17–20% of the human genome, having evolutionarily colonized the genome via a copy-and-paste “life-cycle” mechanism (Moran et al., 1996; Lander et al., 2001; Boissinot and Sookdeo, 2016). Over 99% of germline L1s contain inactivating mutations, with an evolutionarily youngest subfamily, L1HS, retaining retrotransposition competency via ∼80–100 members (Brouha et al., 2003). Germline L1s can also vary among individuals and populations (Rangwala et al., 2009; Rouchka et al., 2010).

Classical cell culture studies have demonstrated that full-length L1 mRNAs are bicistronic, containing ORF1 and ORF2 on a single mRNA (Dombroski et al., 1991; Mathias et al., 1991; Feng et al., 1996; Kaer et al., 2011; Deininger et al., 2016; Naufer et al., 2019). ORF1 is a trimer-forming RNA–binding protein and ORF2 contains functional RT and endonuclease (EN) domains (Mathias et al., 1991; Feng et al., 1996; Rangwala et al., 2009; Naufer et al., 2019). These proteins act in *cis* to facilitate L1 retrotransposition—ORF1 trimers bind to L1 mRNA, forming a ribonucleoprotein complex, after which L1 mRNA is reverse transcribed to produce a single-stranded L1 cDNA (via the ORF2 RT domain) followed by insertion into the genome (via the ORF2 EN domain; Moran et al., 1996). Additionally, in *trans*, ORF2 can reverse transcribe non-self RNA, which has been proposed to generate germline processed pseudogenes, ALUs/SINEs (human repetitive elements), and single-stranded DNA (ssDNA), without requiring a functional ORF1 (Feng et al., 1996; Dewannieux et al., 2003; Gilbert et al., 2005; Garcia-Perez et al., 2007; Wei et al., 2019). ORF2 structures showed distinct RT and EN domains, facilitating the generation of DNA double-strand breaks (DSBs) in the absence of a functional RT domain and cytosolic RNA:DNA hybrids in the absence of a functional EN domain, underscoring ORF2 functional potential beyond L1 retrotransposition and the possible functionality of truncated species (Baldwin et al., 2024; Thawani et al., 2024). Foundational L1 studies in the developing mouse nervous system reported somatic retrotransposition of L1 into genomes of proliferating neural progenitor cells (NPCs) and somatic L1 sequences have been detected in adult human brain by short-read sequencing, contributing to SGM and cell diversification, aging, and neurodegeneration including AD and Parkinson's disease (Muotri et al., 2005; Baillie et al., 2011; Coufal et al., 2011; Evrony et al., 2012; Erwin et al., 2014; Richardson et al., 2014; Upton et al., 2015; Guo et al., 2018; De Cecco et al., 2019; Pfaff et al., 2020; Macciardi et al., 2022).

Remaining knowledge gaps include brain-wide RT enzymatic activity and cellular sources in aged normal and diseased human brain. Similarly, actual L1 transcriptomic sequences and their functional variability in human brain settings are not known, since virtually all previous sequencing studies have relied on short-read sequencing, which cannot capture the entire L1 transcript. Here we assessed actual RT activity and endogenous L1 sequences by PacBio long-read sequencing within the postmortem nondiseased (ND) and AD human cerebral cortex (46–94 years) and their cellular relationships, revealing pervasive RT activity and unexpected ORF2 transcriptomic expression associated with neurons, supporting ORF2 RT and EN functions beyond full-length L1 retrotransposition in the human brain.

## Materials and Methods

### Human postmortem brain tissues

Frozen human brain tissue samples from Brodmann area (BA) 8/9 (prefrontal cortex, PFC) and BA 21 (medial temporal gyrus, MTG) were obtained from multiple brain banks and stored at −80°C. Brain bank tissues sources included: Dalhousie, Emory University, Neurobiobank (Sepulveda), Southwest Dementia Brain Bank, University of California San Diego ADRC, and Washington University. Samples were sectioned in a −20°C cryostat with serial sections taken: six 10 μm sections for RNAscope, three 100 μm sections for RT activity analysis and PacBio Iso-Seq, and one 20 μm section for RNA integrity number (RIN) measurement. Age, sex, postmortem interval, and RIN-matched samples were selected from ND and AD donors from prefrontal (*n* = 8;8) and temporal cortices (*n* = 8;7; Extended Data Table 1-1; Extended Data Fig. 1-1*H*–*J*).

### Cell culture

LN229s were originally purchased from ATCC. Cells were maintained in Dulbecco's modified Eagle's medium containing 5% fetal bovine serum and 100 U/ml penicillin–streptomycin at 37°C under 5% CO_2_. Cell line authenticity was confirmed via short tandem repeat profiling, and cells were confirmed mycoplasma free via the InvivoGen MycoStrip Mycoplasma detection kit.

### RNA ISH: ACD RNAscope

To detect single mRNA molecules, RNAscope was performed on fresh-frozen ND and AD PFC and MTG sections. Ten micrometer sections were cut from frozen biopsies, mounted on Superfrost Gold Plus slides, dried for 1 h at −20°C, and stored at −80°C. In this study, one 3-plex negative control probe [DapB; Advanced Cell Diagnostics (ACD), catalog #320871] and three different probes against genes of interest were used. Each set of experiments included a negative control slide to check probe signal versus background. In situ hybridization (ISH) was performed according to the manufacturer's protocol for RNAscope Multiplex Fluorescent Reagent Kit v2 (ACD, catalog #320293) with minor modifications.

Briefly, dried slides from frozen brains were incubated in cold 4% PFA for 15 min. Slides were then dehydrated in 50, 70, and 100% (two times) ethanol for 5 min each at room temperature. After drying the slides for 5 min at room temperature, hydrophobic barriers were added to reduce reagent use and allowed to dry. H_2_O_2_ was added for 10 min at room temperature and then washed two times with 1× PBS at room temperature. For antigen accessibility, slides were treated with Protease IV for 10 min at room temperature. C3 and C2 probes were diluted in C1 probes at a 1:1:50 ratio and incubated on the slides for 2 h at 40°C. Signal was amplified according to the protocol. C1 probes were detected with Opal 520 (Akoya, FP1487001KT), C2 probes with Opal 570 (Akoya, FP1488001KT), and C3 probes with Opal 650 (Akoya, FP1496001KT). Before mounting the slices, DAPI was added to label the nuclei. Coverslips were then mounted with Prolong Gold Antifade Mountant (Invitrogen, P36930) and allowed to dry at room temperature overnight. Slides stored at 4°C and imaged within 2 weeks of processing.

RNAscope probes (gene; zz-probe #; target region (bp); catalog #):
MAP2; 20; 3,996–5,120;415721-C3
L1 ORF1; 18; 4–1,014; custom
L1 ORF2; 20; 1,194–2,208; custom

### RNA extraction

Total cellular RNA was isolated from 100 µm sections of the human postmortem brain tissue using the RNeasy Mini Kit (Qiagen) and subjected to RNase-free DNase treatment (Qiagen) for 15 min at room temperature. RNA quality was assessed during initial sample selection using 20 µm sections on an Agilent 4,200 TapeStation, with only samples with a RIN >6 utilized to control for tissue integrity.

### Twist probe design

Twist used the CATCH algorithm (Metsky et al., 2019) to design probes recognizing the 146 full-length L1 sequences (containing both an intact ORF1 and ORF2) identified in hg38 (Penzkofer et al., 2017) and 86 HERVk sequences identified as containing an intact gag, pro, pol, and/or env region. The CATCH algorithm reduces the complexity of common sequences while maintaining full probe capture coverage to them. Probes in the panel tolerate ∼10 mismatches per probe to capture the sequence diversity while minimizing the number of probes to capture them. To optimize capture of long fragments, we designed probes in intervals of ∼500 bp. Probe sequences are the property of Twist Bioscience.

### Library preparation for long-read sequencing

cDNA from RNA was prepared using NEBNext Single Cell/Low Input RNA Library Prep Kit for Illumina (E6421), which utilizes poly-DT primed cDNA synthesis to enrich for polyadenylated transcripts. Fragment analysis of cDNA revealed average peak lengths of 2,402 bp (mean, SD 297 bp), which is in keeping with previously reported average transcript length of human cortical mRNA via PacBio Iso-Seq (2–3 kb in length, mean length of 2.46 kb) and corresponding to the mean length of mRNA in the human genome (Piovesan et al., 2019; Leung et al., 2021). Total RNA (130–200 ng) was used for cDNA synthesis followed by 14 cycles of cDNA amplification using Platinum SuperFi II DNA polymerase, which has a high-fidelity rate (>300× Taq fidelity) and is capable of efficiently amplifying DNA with AT content of up to 90%, a critical component given L1's AT-rich sequence. Libraries were then enriched for L1 and HERVk sequences using custom-designed probes from Twist Bioscience and the Twist Standard Hyb and Wash Kit v2 (Twist Bioscience, 104446) followed by 24 cycles of post-pulldown amplification. Samples were cleaned up with 1.3× ProNex beads (Promega, catalog #NG2001). After purification, amplified cDNA went into the SMRTbell library construction according to the protocol: “Preparing SMRTbell libraries using PacBio barcoded overhang adapters for multiplexing amplicons” (PacBio, catalog #PN-101-791-700). Primer annealing and polymerase binding was performed using the Sequel II binding kit 2.0 (PacBio, catalog #PN-101789500), and samples were barcoded to allow for sequencing of four samples per SMRTcell. Finally, the samples were sequenced on Sequel II using Sequel II Sequencing Plate 2.0. An average of 2.8 million polymerase reads were obtained per SMRTcell, and ∼23 million long reads were obtained in total. Sequences were then processed to isolate ∼16 million high-quality reads (72.3% of reads; Wenger et al., 2019).

### Long-read sequencing quality control

Long reads obtained from the PacBio Sequel II were used to generate high-quality consensus reads using ccs (v6.4.0) and –min-rq 0.9. Barcoded adapters were removed, and proper read orientation was determined using lima (v2.6.0). Isoseq3 refine (v3.7) was used to generate full-length nonconcatemeric reads.

### Censor identification of TEs

Full-length, nonconcatemeric reads were used as the input to Censor (v4.2.29) using the censor.ncbi script and ncbi-blast (2.2.9) with the provided human reference library of repeats (perl censor.ncbi SAMPLE_flnc.fasta -lib hum; Kohany et al., 2006).

### Alignment with consensus L1 and HERVK

Full-length, nonconcatemeric reads were aligned to the consensus L1 sequence (Brouha et al., 2003) and consensus HERVk sequence using minimap2 (v2.17-r941) using the parameters -ax splice (to allow for large mid-sequence deletions) and –cs = long. Reads identified as containing L1 in this manner were identified as “L1-containing sequences” and “HERV-containing sequences” (Extended Data Dataset 1-1).

### Protein-coding variant identification

L1-containing sequences were uploaded to the Galaxy web platform, and the public server at usegalaxy.org was used to analyze the data (The Galaxy Community, 2022). Open reading frames within L1-containing sequences were identified via GetORF (Rice et al., 2000; Blankenberg et al., 2007). Identified ORFs were then aligned to consensus ORF1 and ORF2 sequences from UniProt (Bateman et al., 2023) [Q9UN81; O00370] via BLASTp (Altschul et al., 1997; Cock et al., 2015), allowing L1-containing transcripts to be assigned into subcategories: no intact ORFs, ORF1 + ORF2, monocistronic ORF1, monocistronic ORF2, partial ORF2 (ORF2P), and ORF1 + ORF2P. Variants were called if they were supported by ≥3 reads.

### Flanking region, YY1, and 5′-UTR analysis

To investigate whether the expressed elements contained an intact YY1-binding site, we quantified the number of L1-containing sequences with an exact match to the YY1-binding motif (CAAGATGGCCG) via blastn in the Galaxy web platform. A similar approach was taken in identifying the number of 5′-UTR containing L1-containing sequences. L1 masked reads were aligned via blastn in the Galaxy web platform to GRCh38.p14 to identify non-L1 flanking regions mapping to the human reference genome. Reads were categorized as intragenic if their flanking regions mapped within the coordinates of annotated genes or intergenic if their flanking regions mapped elsewhere in the genome. Other reads remained uncategorized because their flanking regions did not map confidently or they did not contain flanking regions.

### Identification of fragmented cDNAs

Non-L1 and non-HERVk–containing reads were examined to determine the level of fragmentation present in the cDNA libraries. Full-length, nonconcatemeric reads were clustered using isoseq3 cluster (v3.7) and mapped to the GRCh38 reference genome using minimap2 (v2.17-r941) -ax splice -uf –secondary = no -C5. Redundant isoforms were collapsed using cDNA_Cupcake (v29.0.0; https://github.com/Magdoll/cDNA_Cupcake). The isoforms were classified and filtered using SQANTI3 (https://github.com/ConesaLab/SQANTI3), and the number of reads associated with incomplete-splice match and full-splice match isoforms was extracted.

### Identification of genomic l1 variants in hg38

L1-annotation bed files for the human reference genome (hg38) were downloaded from Repeatmasker via UCSC. Bam files were uploaded to the Galaxy web platform, and the public server at usegalaxy.org was used to analyze the data. Reads were then extracted via bedtools getfasta (Quinlan and Hall, 2010), and open reading frames were identified via GetORF. Identified ORFs were then aligned to consensus ORF1 and ORF2 sequences from UniProt [Q9UN81; O00370] via BLASTp, allowing assignment and quantification as bicistronic (ORF1 + ORF2, ORF1 + ORF2P) and monocistronic (ORF2 alone, ORF2P alone, and ORF1 alone) L1 sequences.

### Multiple sequence alignment (MSA)

Single amino acid variants (SAVs) were identified by aligning identified ORF2 sequences (codon-based amino acid sequences) with the ORF2 sequence from the L1 consensus (Brouha et al., 2003) via BLAST. Alignments were then uploaded and visualized via the NCBI MSA viewer and annotated to show differences compared with consensus.

### Fluorescence product-enhanced RT (FPERT) assay

FPERT assay was adapted from Ma and Khan (2009). Duplicate human brain tissue sections (100 µm) or triplicated cell transfections were homogenized and incubated for 30 min on ice in RT lysis buffer (RTLB) containing 25 mM Tris, 50 mM KCl, 0.25 mM EDTA, 50% glycerol, 0.5% Triton X-100, 5 mM DTT, and 1× cOmplete, and EDTA-free protease inhibitor cocktail (Sigma-Aldrich). Lysates were centrifuged at 21,000 rpm at 4°C for 5 min to remove lipid-rich cell debris. Supernatant was then collected, and supernatant protein concentration quantified using Bio-Rad Bradford Protein assay (Bio-Rad, #5000001). Protein lysates were diluted to 1 µg/µl and aliquoted to reduce freeze–thaw cycles. FPERT reaction mastermix was created containing 1× PCR buffer, 3 mM MgCl_2_, 0.26 mM dNTPs, 0.6 mM DTT, 0.1 U/µl RNase OUT, 0.085% NP-40, primer/probe mix, and annealed PrimerA/MS2 RNA. A 1.6 µg of protein lysate was added to 53.4 µl of the FPERT cocktail and then assayed in quintuplets of 10 µl in a 384-well plate by qPCR in a CFX-384. After brief centrifugation of the plate, the reaction was carried out according to the following program:

RT reaction: 45 min at 37°C
Polymerase activation: 5 min at 95°C
Amplification: 50 cycles of 5 s at 95°C; 5 s at 60°C; 15 s at 72°C

Serial dilutions of recombinant HIV-1 RT protein (Abcam #AB63979-1001) at concentrations of 10^2^–10^7^ pU were run in parallel in each assay and values were extrapolated from the obtained Cq values, with 40 cycles as the cutoff for background. Heat inactivated lysates (15 min at 70°C) were utilized as background controls and RTLB as no RT, negative controls:

MS2 Primer a: GCC TTA GCA GTG CCC TGT T
MS2 Primer b: AAC ATG CTC GAG GGC CTT A
MS2 probe: /56-FAM/CCC GTG GGA T/ZEN/G CTC CTA CAT GTC A/3IABkFQ/

### Plasmids

ORF2 variants (Fig. 5) were synthesized and inserted into pTWIST CMV PURO expression vectors (high copy number) using Twist gene synthesis services (Twist Bioscience). Sequence accuracy was confirmed via NGS. The 12 variant sequences can be found in Extended Data. Known RT genes (ORF2, HERVk-pol, and H-TERT) were acquired from commercial or public sources:

1. *PBUD-ORF2-CH-3xFLAG*: Original Vector from Addgene—plasmid #51289; Wagstaff et al. (2011). 3xFLAG sequence added to the 3′end using BamHI and EcoRI, removing His-Myc tag.
2. *PBUD–HERVk-con-pol-3xFLAG*: HERVk-con sequence from Lee and Bieniasz (2007); synthesized by Twist Bioscience with Kozak and HindIII on the 5′ end and BamHI-Stop codon-EcoRI on the 3′ end. Sequence optimized to remove 2 EcoRI and HindIII sites within HERVk-con sequence, with no modifications to the amino acid sequence. Cloned into pBUD backbone with 3xFLAG at the 3′ end.
3. *PBUD–HTERT-3xFLAG*: HTERT clone from Dharmacon, BC172541. Cloned into the pBUD backbone using ClaI and XbaI RE digest, 3xFLAG added to 3′ end.

### Transient cell transfections

LN229s were seeded in 12-well plates for RT activity assessments and 8-well chamber slides for examination of EN activity via γ-H2AX. At 70–80% confluency, cells were transiently transfected with Lipofectamine LTX PLUS according to protocol (Thermo Fisher Scientific, #A12621). A PCXN2.1_EGFP construct was utilized as a control. Cells were collected (plates) or fixed (chamber slides) for downstream analyses 24 h post-transfection, with transfection efficiency of ∼75% confirmed in EGFP-positive cells via Cell Countess.

### Immunocytochemistry (ICC)

Cells were plated on cell culture-treated slides, transfected 24 h later and then fixed 48 h post plating using 10% NBF for 5 min at room temperature. Samples were permeabilized with PBS + 0.1% Triton X-100 for 15 min and then blocked using DAKO Universal Antibody Diluent for 1 h (Agilent #S302283-2). Slides were incubated with anti-phospho-histone H2A.X (Ser139) antibody, clone JBW301 primary antibody (Millipore Sigma #05-636-I; 1:250) overnight in DAKO + 0.1% Tween 20. Slides were washed three times for 10 min with PBS + 0.1% Tween 20. Alexa Fluor 647 anti-mouse secondary antibodies (Thermo Fisher Scientific #A-21237; 1:10,000) were applied at 1:1,000 dilutions in DAKO + 0.1% Tween 20 and incubated at room temperature for 1 h before being washed as previously described. Samples were mounted in VECTASHIELD HardSet Antifade Mounting Medium with DAPI (Vector Laboratories #H-1500) and imaged on a Keyence BZ-X810 at 20×.

### Image acquisition and preprocessing

Entire tissue images were acquired to examine global patterns using a Keyence BZ-X810 at 10×. For all imaging experiments, exposure settings were established during the first acquisition, with thresholds based on signal intensity in negative controls, and not modified afterward. Images for single-cell RNA expression analysis were acquired using a Zeiss microscope at 20× objective. For tissue sections, five regions from cortical sections were selected at random and *z*-stacks collected (5; 1.4 µm interval). For quantification of signal intensity, conventional fluorescence was utilized with *z*-stacks collapsed into the orthogonal projection. Ten images per well (with biological triplicates for each condition) for ICC analysis were acquired using a Keyence BZ-X810 at 20×.

### RNAscope and ICC quantification

**Figure 1.**
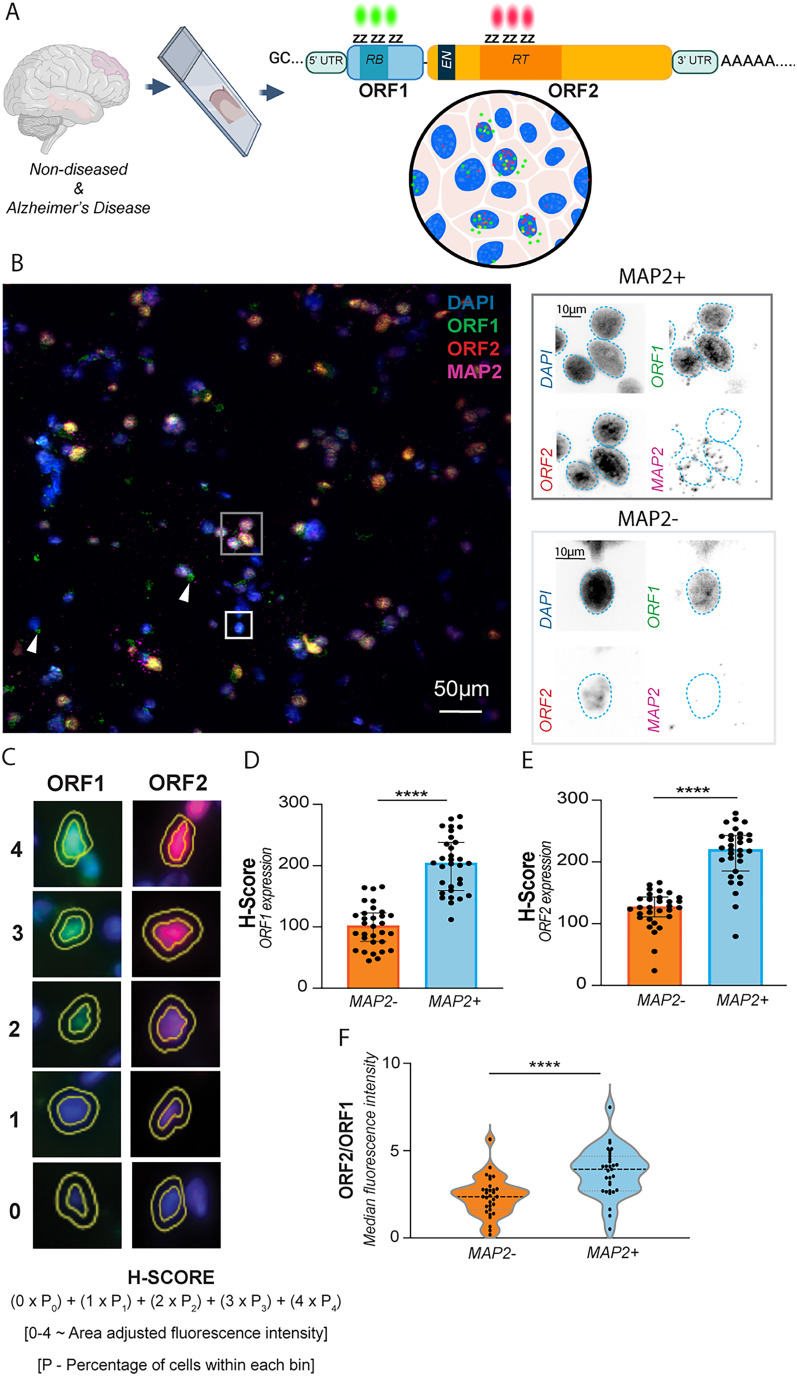
Significant and discordant L1 ORF2 and ORF1 RNA expression in aged human cortical neurons. ***A***, Schematic of RNAscope probes designed against the functional domains of L1 ORF1 and ORF2 for examination of L1 spatial transcriptomic expression in the aged human postmortem brain tissue (ND and AD PFC and MTG, *n* = 31). ***B***, RNAscope of L1 ORF1 (green) and L1 ORF2 (red) show moderate colocalization (yellow) in both MAP2+ (magenta) and MAP2− nuclei. Scale bar, 50 µm. Cytoplasmic ORF1 alone was also detected (green, some noted by white arrowheads). Magnification of MAP2+ and MAP2− nuclei with each probe isolated (gray boxes). Nuclear boundaries determined by DAPI labeling (dashed blue line). ***C***, H-score calculation based on the percentage of cells binned according to probe signal intensity. Cells were scored from 0 to 4 based on area-adjusted MFI as described in methods and prior literature (Jolly et al., 2019); 0–400; 0, no-signal; 400, highest signal. ***D***, ORF1 H-score for MAP2− cells compared with MAP2+ cells. Median ± interquartile range (IQR). ****p < 0.0001. Wilcoxon test. ***E***, ORF2 H-score for MAP2− cells compared with MAP2+ cells. Median ± IQR. ****p < 0.0001. Wilcoxon test. ***F***, A violin plot of ORF2/ORF1 MFI ratio per individual MAP2− versus MAP2+ cells. Median ± IQR. ****p < 0.0001. Wilcoxon test.

The QuPath analysis software was utilized to identify discrete cells based on DAPI nuclear stain (Bankhead et al., 2017; Jolly et al., 2019; Extended Data Fig. 1-1). For RNAscope in human brain sections, cell types were then identified due to the absence or presence of >3 puncta of the cell-specific markers (MAP2). Median signal intensity for ORF1 and ORF2 was quantified per cell, controlling for the nuclear size and probe number. Background signal intensity was quantified per slide and controlled for to compare between samples. Changes in probe median fluorescence intensity (MFI) were independent of fluorophore (Extended Data Fig. 1-1). ORF1 and ORF2 expression could not be distinguished as discrete puncta, so MFI was quantified to determine the expression level (Bankhead et al., 2017; Jolly et al., 2019; Extended Data Fig. 1-1). MFI was then utilized to determine an H-score for each tissue section, allowing us to take into consideration heterogeneous expression across cells in a tissue (Fig. 1*C*).

In functional assays examining γH2AX, nuclei were identified based on DAPI signal and sum signal intensity/area quantified per cell for γH2AX. Cells were quantified across triplicate experiments, with 10 images per experiment. Fold change was calculated for each cell based on the median signal intensity/area for all cells in EGFP control, normalizing EGFP control to 1 (increased expression >1, decreased expression <1). We quantified 8,201–13,420 nuclei per condition.

### Statistical analyses

Data analyzed utilizing statistical tests noted in the results based on normality of the data and singular versus multiple comparisons. Statistical analyses run in Prism 10.0.1.

### Figures

Figures were created with the aid of BioRender, Adobe Illustrator, and Prism.

### Data availability

Bam files for PacBio reads are available through the European Genome-Phenome Archive (EGA) with accession number EGAS50000000184. For privacy reasons, these data are access controlled. Requests for access can be made through EGA and will be approved upon completion of a Data Access Agreement.

## Results

### L1 ORF1 and ORF2 RNAs are significantly and discordantly expressed in human cortical neurons

ISH of frozen human cortical tissue sections (*n* = 31, AD and ND PFC and MTG, ages 46–94 years) utilized custom-designed RNAscope probes against the functional domains of L1 ORF1 (RNA-binding domain; green) and ORF2 (RT domain, red; Fig. 1*A*; Extended Data Table 1-1). A third probe for neuron-specific MAP2 (magenta) identified neuronal versus non-neuronal (MAP2−) cells (Jolly et al., 2019; Fig. 1*B*). Expressions of both ORF1 and ORF2 were assessed qualitatively and quantitatively via H-score, a method for quantifying the intensity of a fluorescent signal in a cell population that considers both the proportion of cells expressing the marker and the intensity of that expression (Jolly et al., 2019; Fig. 1*B*,*C*). L1 ORF1 and ORF2 probe signals were significantly increased in neuronal nuclei (MAP2+, nuclei denoted by DAPI), a result that was corroborated by increased signal in gray versus white matter (Fig. 1*B*,*D*,*E*; Extended Data Fig. 1-1*A*). Remarkably, individual neurons showed statistically significant discordant ORF2::ORF1 expression compared with non-neurons, with higher ratios of ORF2::ORF1 and spatially nonoverlapping signals, a result that was maintained when reversing fluorophores (Fig. 1*F*; Extended Data Fig. 1-1*B*,*C*), suggesting the presence of monocistronic ORF1 and ORF2 species. Specific signals were absent following RNase treatment, confirming the specificity of probe signal to RNA, rather than DNA, copies of ORF1 and ORF2 (Extended Data Fig. 1-1*D*). Furthermore, cytoplasmic ORF1, but not ORF2, was detected across samples (Fig. 1*B*, white arrows). Neuronal ORF2 expression was slightly decreased in the AD MTG compared with ND MTG (Extended Data Fig. 1*E*–*G*). While targeted against the functional domains of ORF1 and ORF2, RNAscope probes detect both coding and noncoding variants, including intronic L1s found in pre-mRNAs (Deininger et al., 2016; Kaul et al., 2020; Lanciano and Cristofari, 2020). Therefore, additional technologies are necessary to identify the diversity and prevalence of expressed coding bicistronic L1 and monocistronic ORF1 and ORF2.

### Baseline characteristics of L1 sequences in poly(A^+^) mRNA

**Figure 2.**
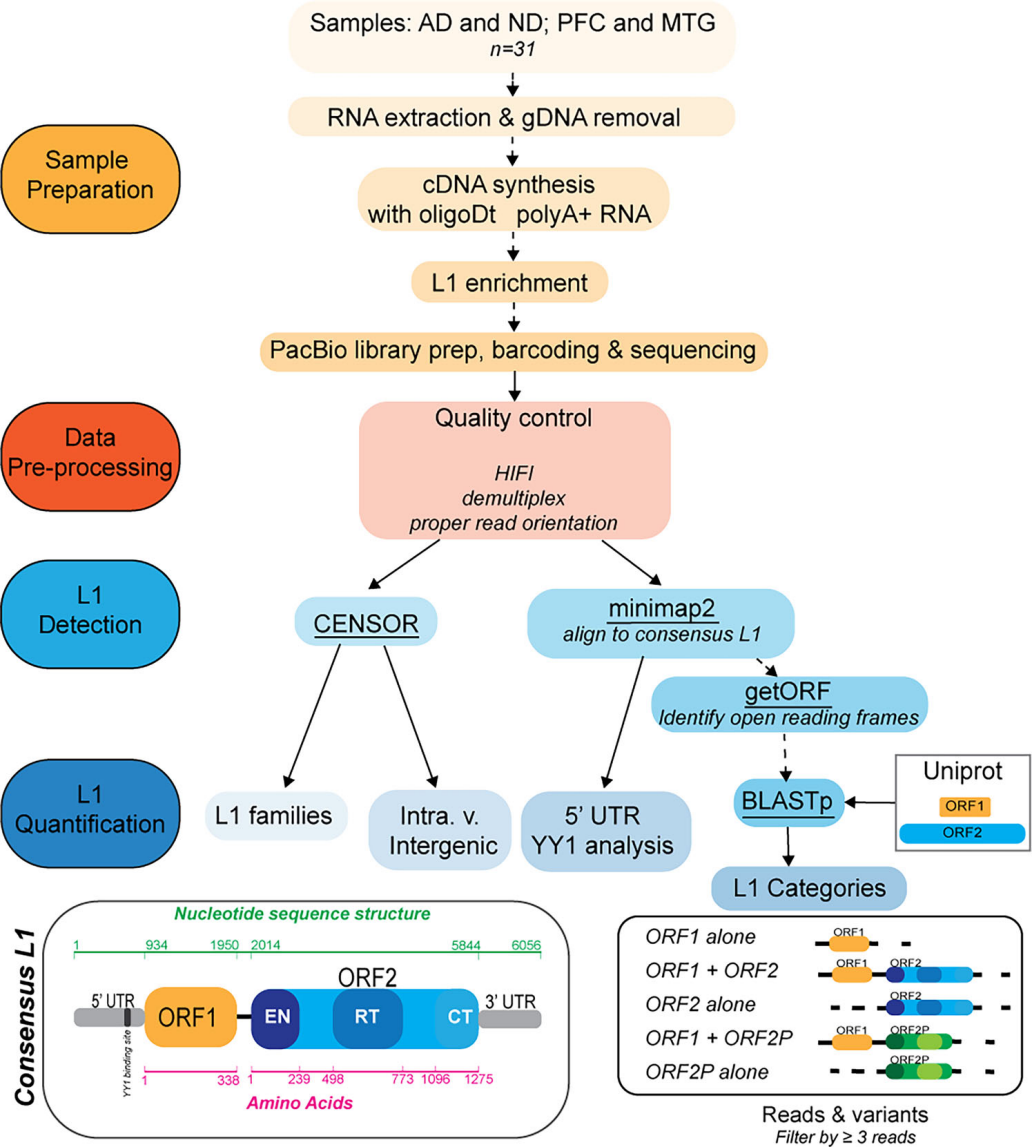
Overview of the experimental and bioinformatic pipeline for PacBio long-read sequencing and analysis of the L1 neural transcriptome. Schematic of the experimental workflow and bioinformatic pipeline for analysis of the poly(A^+^) L1 neural transcriptome. **Sample preparation**: PFC and MTG of ND and AD postmortem human brain (ages 46–94 years; *n* = 31). RNA extracted and gDNA contamination removed through DNase treatment and columns. cDNA synthesized with oligoDT primers to isolate poly(A^+^) RNA transcripts. Library enriched for L1 through custom Twist Bioscience pulldown probes. L1 enriched libraries barcoded to allow sample identification and then sequenced on PacBio Sequel II. **Data pre-processing**: ∼23 million long reads obtained from the PacBio Sequel II were used to generate high-quality consensus reads. Barcoded adapters were removed, and proper read orientation was determined to generate full-length nonconcatemeric reads (∼16 million high-quality reads; 72.3%). **L1 detection**: L1 detected via dual methods—Censor (identification, annotation, and masking via the human reference library of repeats) and minimap2 alignment to the consensus L1 sequence to ID “L1+ sequences” (Brouha et al., 2003). **L1 quantification**: Censor annotation and masking utilized to identify L1 families within each read and the presence of flanking regions mapping back to the reference genome (“read-through” transcripts). L1-containing transcripts examined for prevalence of promoter and regulatory regions. Open reading frames identified via getORF and then aligned to consensus ORF1 and ORF2 sequences from UniProt [Q9UN81; O00370] via BLASTp, allowing L1+ transcripts to be assigned into subcategories: ORF1 alone, ORF1 + ORF2, ORF2 alone, ORF1 + partial ORF2 (ORF2P), and ORF2P alone. Variants called if supported by ≥3 reads.

RNA for sequencing was isolated from adjacent tissue sections of the same 31 AD and ND human postmortem PFC and MTG samples utilized in the RNAscope experiments. cDNA libraries were constructed using oligoDT primers and prepared for PacBio HiFi long-read sequencing, which can generate long-read DNA sequences of up to tens of kilobases in length with 99.9% sequence accuracy, further allowing resolution of complex and AT/GC-rich sequences and identification of structural variants (Charnaud et al., 2022; Logsdon et al., 2020; Fig. 2). Mapping the publicly available PacBio AD bulk Iso-Seq dataset against an L1 consensus sequence (Brouha et al., 2003) revealed that L1 sequences comprise ∼2.56% of the neural transcriptome.

**Figure 3.**
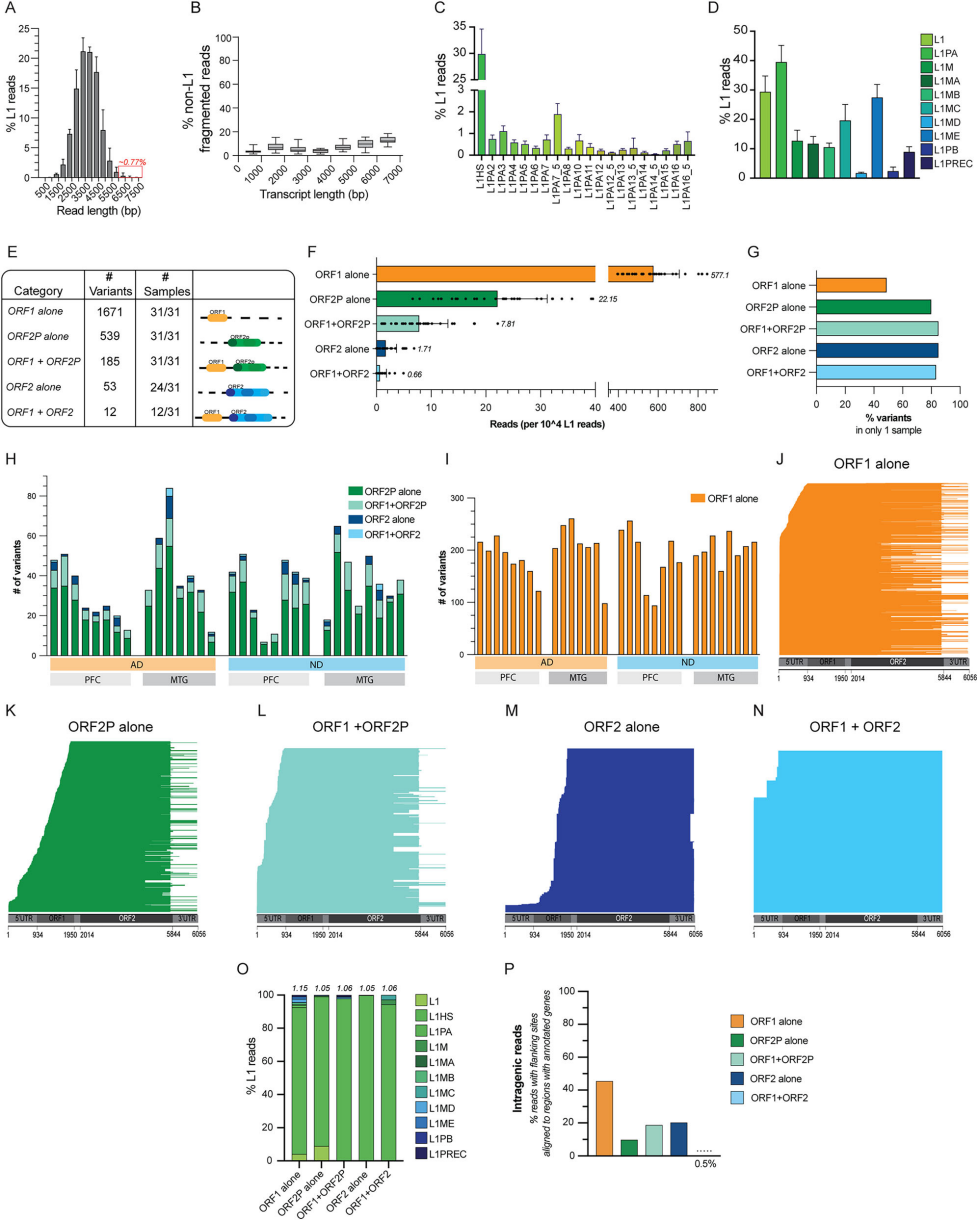
PacBio long-read sequencing of the L1 neural transcriptome reveals interindividual and sample variation. ***A***, Histogram of the percentage of L1 reads of different lengths (bp-basepairs). Bin 500 bp. Median ± interquartile range (IQR). The red square indicates L1 reads ≥6 kb (∼0.77%). ***B***, The percentage of fragmented reads for transcripts of each length based on assessment of captured non-L1 and non-HERVk containing reads as incomplete-splice matches via SQANTI3. Median, IQR, min, and max. ***C***, The percentage of L1 reads assigned to the youngest subfamily of L1, L1PA. Mean + SD. ***D***, The percentage of L1 reads assigned a L1 family annotation based on Censor. Mean + SD. Reads were frequently annotated with multiple subfamilies (average of 1.86 annotations/read)—percentages of read assignments total to >100%. ***E***, The number of variants identified and number of samples containing at least one variant per category. ***F***, Number of reads (per 10,000 L1 reads) identified as containing ORF1 alone, partial ORF2 (ORF2P) alone, ORF1 + ORF2P, ORF2 alone, or ORF1 + ORF2. Dots represent individual samples. Numbers indicative of mean. Error bars indicate SD. ***G***, The percentage of variants identified in only one sample, indicating high interindividual variability in variant expression. H + I. The number of variants for categories (***H***) ORF1 + ORF2, ORF2 alone, ORF1 + ORF2P, ORF2P alone, and (***I***) ORF1 alone per individual sample for AD versus ND samples and PFC versus MTG samples. ***J*–*N***, Pile-ups of reads for ORF1 alone (***J***), ORF2P alone (***K***), ORF1 + ORF2P (***L***), ORF2 alone (***M***), and ORF1 + ORF2 (***N***) as aligned to the L1 consensus sequence. ***O***, The percentage of L1 coding categories (ORF1 alone, ORF2P alone, ORF1 + ORF2P, ORF2 alone, ORF1 + ORF2) reads assigned each L1 family annotation based on Censor. The average number of annotations per read for each coding category noted above each column. ***P***, The percentage of reads within each L1 coding category with flanking regions that aligned to regions of hg38 with annotated genes, indicative of intragenic, “read-through” L1 transcripts.

To obtain greater sequencing depth of L1 sequences specifically, custom Twist Bioscience pulldown probes were used, resulting in a ∼10-fold enrichment of L1-containing reads (Extended Data Fig. 2-1*A*). Alignment of sequences against an L1 consensus sequence identified L1-containing PacBio HiFi reads with a mean read length of 3,541 bp (Fig. 3*A*; Extended Data Fig. 2-1*B*). Only ∼0.77% of L1-containing reads were ≥6 kb, which was nonetheless consistent with virtually undetectable full-length L1 reported in the human brain, as previously determined by the Northern blot (Belancio et al., 2010).

To rule out possible preparative fragmentation as an artifactual source of truncated, smaller L1 variants, we assessed non-L1 and non-HERVk containing reads for annotation as incomplete-splice matches via SQANTI3 (Tardaguila et al., 2018). Minimal fragmentation was identified, indicating that the truncated L1 reads originated from expression of genuine truncated sequences rather than preparative artifact (Fig. 3*B*). L1 reads had significant sense strand enrichment, supporting active L1 transcription (74.55% of reads; ****p < 0.0001, paired *t* test; Pabis et al., 2023). On average, 45.28% of L1 reads within a sample included a 5′-UTR, which contains both the internal RNA polymerase II promoter and important regulatory sites for L1 repression, such as a YY1-binding site (Athanikar et al., 2004). The YY1-binding region is critical for the methylation and regulation of L1; sequences missing YY1-binding motifs are more likely to be expressed via repression evasion (Athanikar et al., 2004). On average, 19.38% of L1 reads within a sample contained an intact YY1-binding region, consistent with active L1 expression and L1 repression evasion.

Using the bioinformatics tool Censor, L1 read regions were masked, allowing for identification and mapping of flanking sequences of coding and noncoding L1 transcripts (Kohany et al., 2006; Fig. 2). Intragenic versus intergenic reads were thus categorized based on the presence or absence of flanking regions mapping back to annotated genes. Intragenic L1 sequenced with non-L1 annotated flanking regions were thus interpreted as expressed via read-through transcription within the introns of pre-mRNA or UTRs of mRNAs (Swergold, 1990; Kaer et al., 2011; Deininger et al., 2016; Pabis et al., 2023). A 43.3% of L1 reads had flanking sites that aligned to regions with annotated genes, indicating that they were intragenic and likely coexpressed with another gene. The remaining L1 sequences either had no flanking sequences/unmappable flanking sequences (51.8%) or flanking sequences that mapped to intergenic regions of the genome (4.9%) and were likely expressed from their own promoter.

### L1 sequences of young L1s were most prevalent in the L1 transcriptome

Quantification of L1 subfamilies via Censor revealed that a majority of individual L1 reads were annotated for multiple L1 subfamilies, with an average of 1.86 subfamily annotations per read. L1PA was most common (39.22%), principally composed of L1HS, the youngest and most active L1 subfamily (Brouha et al., 2003; Garza et al., 2023; Fig. 3*C*). Other reads were annotated as either uncategorical L1 or belonging to other evolutionarily older subfamilies (Fig. 3*D*). Multiannotation of single L1 reads, easily detected by PacBio long-read sequencing, would be missed by short-read RNA-seq or qPCR, likely resulting in inappropriate quantification and identification of L1 reads, with fragmented reads leading to overestimation of different families from a single read.

### Diverse and truncated protein-encoding L1 RNA sequences are prevalent, with a near absence of full-length coding L1

PacBio long-read sequencing enables assessment of intact and contiguous L1 ORF1 and/or ORF2 transcripts, detecting annotated as well as non-annotated L1 variants, including those that may arise from somatic events. To examine the characteristics of different types of L1 variants, we categorized our L1 transcripts based on their protein-coding potential. Open reading frames defined by start and stop codons were identified in L1 transcripts, and the predicted amino acid sequences were aligned with consensus ORF1 and ORF2 amino acid sequences [Uniprot Q9UN81; O00370] (Fig. 2). L1 transcripts were then categorized as ORF1 + ORF2, ORF1 + ORF2P, ORF1 alone, ORF2 alone, or ORF2P alone (Fig. 2). ORF1 + ORF2 describes a bicistronic L1 sequence that contains intact coding sequences for both ORF1 and ORF2. ORF1 + ORF2P is another bicistronic L1 with a 3′-truncated ORF2 sequence that retains the intact RT domain. ORF1 alone, ORF2 alone, and ORF2P alone are all monocistronic L1s that have intact coding sequences for only ORF1 (ORF1 alone), only full-length ORF2 (ORF2 alone), or a truncated ORF2 containing both the EN and RT domain (ORF2P alone). Overall, <0.01% (mean, 0.006%) of reads were full-length coding L1s (containing both intact ORF1 and ORF2 open reading frames), with most brain samples (19/31) lacking any such reads (Fig. 3*E*,*F*). In addition, >80% of L1 sequences were noncoding. Monocistronic transcripts encoding either intact ORF1 or RT-encoding ORF2 were detected in all brain samples, with intact ORF1 representing 5.77% of transcripts and intact ORF2 representing 0.017% of transcripts (mean; Fig. 3*E*,*F*). The more prevalent RT-encoding sequences were L1 ORF1 + ORF2P and monocistronic ORF2P (0.078 and 0.221% of total L1 sequences, respectively; Fig. 3*E*,*F*). Truncated ORF1 and ORF2 transcripts, albeit lacking full-sequence characterization, have been previously reported using Northern blot or 5′-RACE (5′-rapid amplification of cDNA ends) particularly from other tissues (Rangwala et al., 2009; Belancio et al., 2010). These studies examined a range of nonbrain tissues and reported similar percentages of bicistronic intact ORF1 + ORF2, monocistronic intact ORF1, and monocistronic intact ORF2, suggesting tissue-specific differences in the prevalence of expressed L1 species (Rangwala et al., 2009).

### High sample variability of L1 coding mRNAs

Many ORF1 and ORF2 variants were expressed within each brain sample, ranging from the extremely rare full-length bicistronic transcripts to prevalent monocistronic transcripts; however, their actual sequence identity was most often unique to a given sample. Only 12 full-length L1 (ORF1 + ORF2) variants were identified across all samples (Fig. 3*E*). In comparison, 53 intact ORF2 variants were identified (Fig. 3*E*). Bicistronic ORF1 + ORF2P, monocistronic ORF1 alone, and ORF2P alone constituted significantly more variants (*n* = 185; 1,671; 539; Fig. 3*E*). A high degree of sample variability was consistently observed among categories, with 80–85% of ORF2 variants only identified in a single sample (Fig. 3*G*). Contrasting with ORF2 variants, ORF1 alone variants (*n* = 1,671) had less interindividual variability, with only 49.0% of variants expressed in a single sample (Fig. 3*G*). The high percentage of variants only seen in single samples is reminiscent of SGM, encompassing DNA sequence differences among brain cells and brain regions, and further consistent with somatic L1 variability as reported in human lymphoblastoid cell lines (Rangwala et al., 2009; Lee et al., 2018; Kaeser and Chun, 2020; Bae et al., 2022; Pascarella et al., 2022). Neither prevalence of L1 reads within an individual category nor specific L1 variants corresponded with region or disease state (Extended Data Fig. 2-1*C*–*G*).

These data support the ORF2::ORF1 discordance detected via RNAscope as being due to monocistronic ORF1 and ORF2 expression. Importantly, short-read sequencing likely misidentifies monocistronic ORF1 and ORF2 reads as full-length bicistronic L1 transcripts based on common mapping techniques, highlighting the advantages of long-read sequencing technologies for the study of L1. Genomic (hg38) L1 sequences were evaluated with the same criteria, revealing that a paucity of the potential genomic copies of ORF1 + ORF2 variants noted in hg38 are expressed (Table 1). Significantly more ORF2P variants (bicistronic and monocistronic; *n* = 185; 539) were identified within the neural transcriptome than would have been expected based on the reference genome (*n* = 93,173; hg38), indicative of a high degree of L1 transcriptomic variability of unclear etiology within this subcategory.

**Table 1.** Protein-coding sequences identified in genomic (hg38) L1 sequences

hg38	Reads	Variants
*ORF*1 alone	568	553
*ORF*2*P* alone	173	173
*ORF*1 *+* *ORF*2*P*	93	93
*ORF*2 alone	105	104
*ORF*1 *+* *ORF*2	156	154

### 5′- and 3′-truncation of intact L1 ORFs

Monocistronic ORF1 transcripts generally aligned to the full L1 consensus sequence (Brouha et al., 2003), with a subset having 5′-truncation, 3′-truncation, and/or polyadenylation within the consensus ORF2 region, indicating that monocistronic ORF1 protein expression predominantly occurred via mutations and early stop codons within the ORF2 region, rather than significant 3′-truncation (Fig. 3*I*). A significant portion of bicistronic ORF1 + ORF2P, monocistronic ORF2P, and monocistronic ORF1 were truncated at the 5,500–5,800 region, as previously reported (Belancio et al., 2006; Fig. 3*J*–*L*). A significant number of monocistronic ORF2 and ORF2P reads were truncated in the 5′ region and thus did not contain the 5′-UTR internal RNA polymerase II promoter or important L1 regulatory sites (Fig. 3*K*,*M*). Similar 5′-truncations were also seen in bicistronic ORF1 + ORF2 and ORF2 + ORF2P (Fig. 3*L*,*N*).

A majority of reads in each category were annotated as L1HS via Censor (Fig. 3*O*). Notably, potential protein-coding reads showed markedly less multifamily annotation (1.05–1.15 annotations/read) compared with overall L1-containing reads (1.86 annotations/read). Analysis of flanking regions, as determined by masking L1 regions of reads via Censor, indicated that most variants did not have flanking sequences or had flanking sequences that did not map to the genome. While 43.3% of all L1 reads showed flanking sites aligning to the genome, a smaller proportion of coding bicistronic L1 (ORF1 + ORF2 and ORF1 + ORF2P) and monocistronic ORF2 (ORF2 alone and ORF2P) had intragenic flanking regions (0.5, 18.8, 20.3, 9.8%, respectively; Fig. 3*P*). In contrast, 45.4% ORF1 alone reads had flanking regions that mapped within gene coordinates, indicating that these reads were likely intragenic and coexpressed with other genes.

### Endogenous RT activity in the human brain correlates with monocistronic ORF2 variant expression

**Figure 4.**
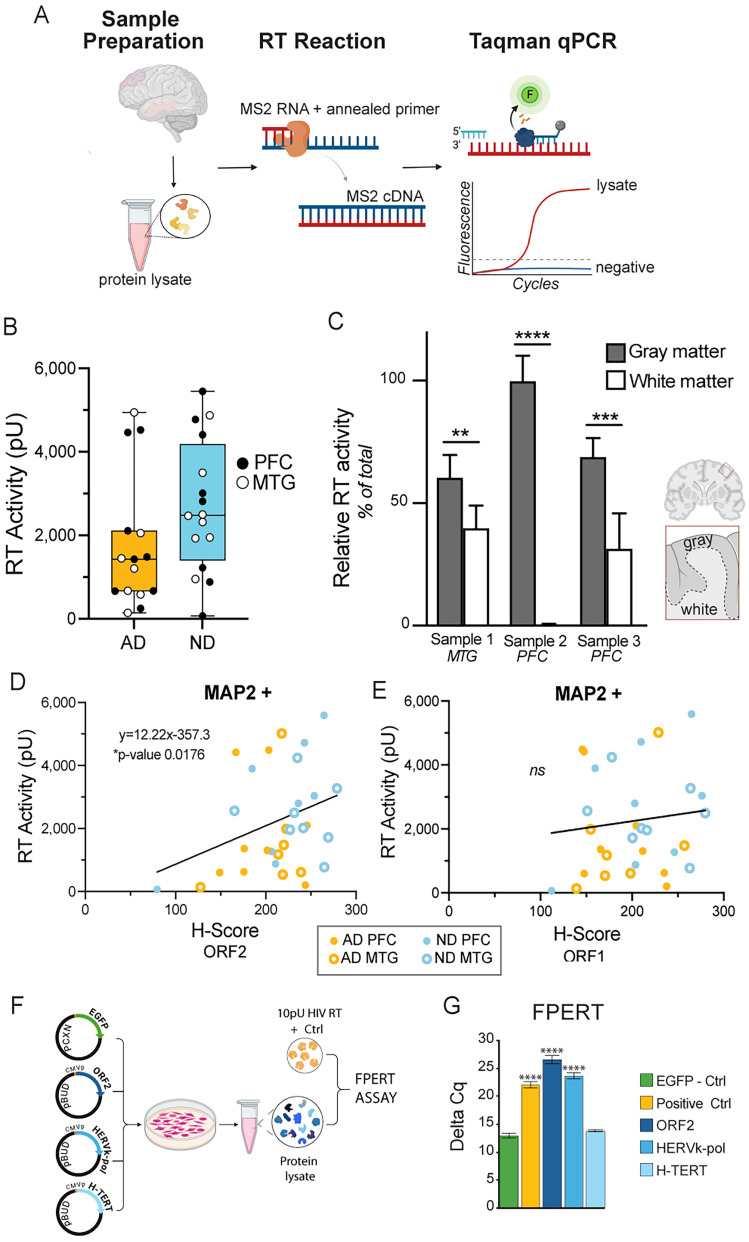
RT activity in the human brain correlates with L1 ORF2 in neurons. ***A***, Schematic of the experimental workflow and FPERT assay. ***B***. RT activity (as pU of control recombinant HIV RT) of postmortem human brain samples from ND and AD PFC (filled circles) and MTG (open circles). Median ± interquartile range (IQR), min and max, nonsignificant (ns): Mann–Whitney test. ***C***, Relative RT activity of the microdissected gray versus white matter compared with whole brain lysates from three brains. Mean ± SD. **p < 0.01; ***p < 0.001; ****p < 0.0001. Multiple unpaired *t* test. ***D***, Scatterplot of RT activity (pU) in postmortem human brain samples (*n* = 31) relative to ORF2 H-score in MAP2 + nuclei. *p = 0.0176; *y* = 12.22 × −357.3; simple linear regression. ***E***, Scatterplot of RT activity (pU) in postmortem human brain samples (*n* = 31) relative to ORF1 H-score in MAP2+ nuclei. *ns.* Simple linear regression. ***F***, Schematic of RT expression vector transfection and activity assessment via FPERT assay. ***G***, FPERT activity assessment of RT protein lysates (ORF2, HERVk-pol, and H-TERT) compared with transfection controls (EGFP—background cell lysate RT activity) and RT activity-positive controls (10pU HIV RT). Delta Cq: negative control Cq (no RT, no signal after 50 cycles)—sample Cq; *n* = 3, mean, SD; one-way ANOVA with Tukey–Kramer; ****p < 0.0001.

To examine the relationship between ORF2 transcriptomic expression and endogenous RT activity, we assayed by FPERT the cerebral cortical protein lysates from tissue sections adjacent to those analyzed by spatial transcriptomics (Ma and Khan, 2009; Fig. 4*A*, 31 brain samples). Virtually all samples had detectable endogenous RT activity, regardless of the brain region or disease state (Fig. 4*B*), with AD samples showing lower RT activity—consistent with neuronal loss associated with terminal AD. To assess endogenous RT activity in regions of high neuronal versus high non-neuronal composition, we microdissected cortical tissue sections for the gray matter (estimated at 1.5:1 neurons:glia) versus white matter (estimated at 1:10 neurons:glia; *n* = 3) and lysates examined for RT activity (von Bartheld et al., 2016). Overall, RT activity was significantly higher in gray compared with white matter in each sample, positively correlating with the increased expression of ORF2 in neurons (Figs. 1*E*, 4*C*). RT activity showed a statistically significant correlation with neuronal (MAP2-positive) ORF2 H-score, but not ORF1 H-score (Fig. 4*D*,*E*), consistent with a major RT activity contribution from neuronal monocistronic ORF2.

Other genomic sources of RT activity beyond ORF2 were also considered, including HERVk-pol and human telomerase RT (H-TERT; Cong et al., 2002; Baldwin et al., 2022). Analyses of HERVk-enriched cDNA libraries using PacBio long-read sequencing detected no intact HERVk-pol mRNAs in any of the brain samples. Furthermore, RT activity produced by L1 and HERVk-pol, but not H-TERT, gene overexpression is detectable and significantly increased compared with controls (gene constructs transiently overexpressed in cell culture and then assessed via FPERT; Fig. 4*F*,*G*). These data strongly support ORF2 and its variants as a major source of neuronal RT activity in the human brain.

### ORF2 transcriptomic variants show highly variable sequences and RT/EN activities

**Figure 5.**
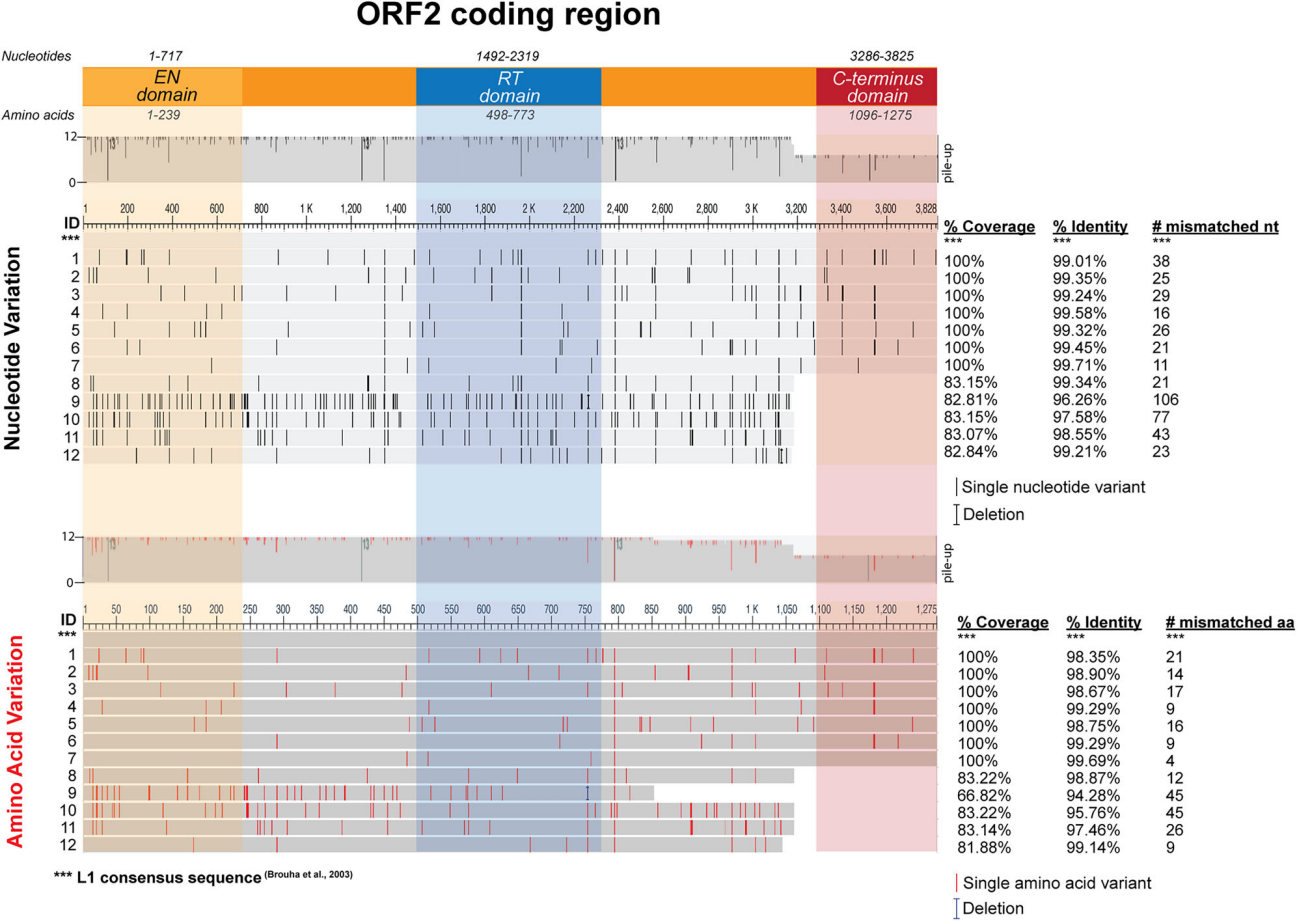
Nucleotide and amino acid variation across L1 ORF2 variants for overexpression-based function assays. Nucleotide and amino acid variants across the 12 functionally assayed ORF2 variants as compared with the ORF2 region in the L1 consensus sequence (Brouha et al., 2003). EN (yellow), RT (blue), and C-terminus (red) domains are highlighted. Percentage identity, percentage coverage, and the number of mismatched nucleotides and amino acids as compared with the L1 consensus sequence.

A remarkable spectrum of predicted amino acid variability was observed across L1 mRNA sequences encoding ORF2 with an intact RT domain as compared with the consensus ORF2 amino acid sequence (Uniprot O00370). A focused analysis of 12 ORF2 coding variants from monocistronic ORF2 transcripts (Fig. 5) identified significant nucleotide sequence variability (Fig. 5, nucleotide variants) that generate productive amino acid differences (Fig. 5; Extended Data Fig. 3-1, amino acid variants), affecting all functional domains of ORF2.

**Figure 6.**
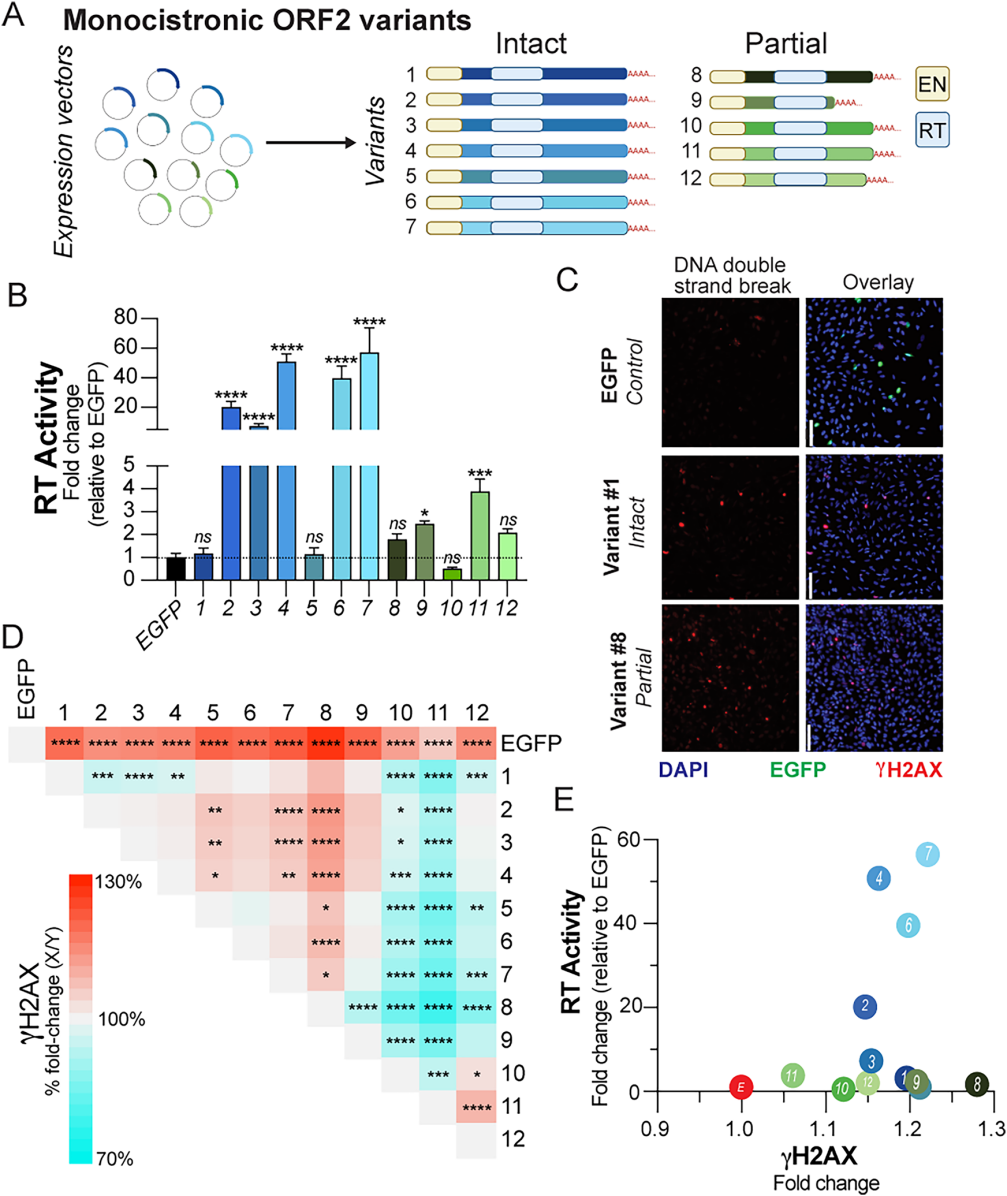
Intact and partial ORF2 variants show concomitant EN and RT activities. ***A***, Twelve isolated protein-encoding ORF2 variants utilized in the functional assays: intact ORF2 variants (blue, #1–7) contain an EN domain (yellow), RT domain (light blue), and C-terminus; partial ORF2 variants (green, #8–12) contain an intact EN and RT domain but a truncated/absent C-terminus; color scheme maintained throughout the figure. ***B***, Fold change in RT activity, as determined by FPERT assay, of ORF2 variants relative to EGFP control transfections across triplicate experiments. Mean ± SEM. *p < 0.05; ***p < 0.001; ****p < 0.0001. One-way ANOVA. ***C***, Transfected LN229s (ORF2 variant expression constructs vs EGFP) were labeled for γ-H2AX (red), a marker of double-strand DNA breaks. Scale bar, 100 μm, 20×. Representative images of EGFP controls, ORF2 variant #1 (intact), and ORF2 variant #8 (partial). ***D***, Heat map of the difference in fold change of γ-H2AX MFI between conditions (*x*-axis minus *y*-axis variants). Asterisks indicate statistical significance of fold change in γ-H2AX MFI/nuclei between individual conditions. *p < 0.05; **p < 0.01; ***p < 0.001; ****p < 0.0001. Kruskal–Wallis test with multiple comparisons. Empty boxes denote a lack of statistical significance. ***E***, Scatterplot of fold change of γ-H2AX MFI versus mean RT activity of variants. Dot color and number indicative of variant ID.

To assess whether endogenous monocistronic ORF2 variants have variable RT and EN activities, we selected 12 variants representative of monocistronic intact and partial ORF2 sequences (ORF2 alone, seven sequences; ORF2P alone, five sequences; Fig. 6*A*; Extended Data Fig. 5-1). These variants were identified in both ND and AD brains, including PFC and MTG, and did not encode an intact ORF1 protein (Extended Data Fig. 5-1). Constructs encoding the 12 isolated ORF2 variants were commercially synthesized, sequence validated and transiently transfected as CMV-promoter expression constructs into the LN229 cell line, controlling for transfection efficiency by concurrent EGFP transfection. Samples were then assayed for RT activity by the FPERT assay with duplicate samples analyzed for EN activity by γ-H2AX labeling in formaldehyde-fixed cell cultures (Kinner et al., 2008).

Seven of the 12 variants showed statistically significant RT activity above the baseline, with a dynamic range of ∼50× over control (Fig. 6*B*; one-way ANOVA, EGFP control Cq vs Variant Cq). Truncated ORF2s also showed RT activity, although at lower levels than those of intact ORF2s (Fig. 4*B*). However, some ORF2 variants, including full-length variants, lacked functional RT activity, despite the presence of an intact coding RT domain. EN activity was assessed by employing γ-H2AX immunolabeling, a robust marker of DSBs in cells and tissues (Kinner et al., 2008). All 12 ORF2 variants showed increased γ-H2AX signals versus the EGFP transfection control, with labeling that varied ∼1.3-fold across the samples (Fig. 6*C*,*D*). Interestingly, γ-H2AX labeling was greatest in a partial variant that lacked RT activity (#8; Fig. 6*E*), suggesting a potential role, independent of RT activity, for partial ORF2 variants with intact EN domains via the generation of DSBs in the brain. A nonlinear relationship between EN and RT activities was observed—the highest burden of DSBs was caused by a variant with low levels of RT activity (#8), while yet another variant demonstrated both relatively high EN and RT activities (#7; Fig. 6*E*).

Recent structural analyses of ORF2 identified several essential amino acid residues required for recognition of the poly(A) tracts. Mutations of these residues decrease target-primed reverse transcription (TPRT) but not EN or RT activities (Baldwin et al., 2024; Thawani et al., 2024). Of the eight amino acids that make hydrogen bonds with the poly(A) tract, six were conserved across all monocistronic ORF2 variants, with mutations for two others being present in a minority of variants (1.69 and 18.64%). As such, a majority of the ORF2 intact monocistronic variants are predicted to be capable of TPRT. By comparison, partial ORF2 variants had substantially more heterogeneity in these regions, with single amino acid variants (SAVs) and truncations leading to substitutions or complete loss of these amino acids. The 12 variants in the functional assay contained a range of SAVs across functional domains (Extended Data Fig. 3-1). RT activity was still produced by even the most truncated predicted protein (#9) that contained numerous SAVs, confirming that loss of poly(A) tract binding does not necessarily eliminate RT activity. Further functional analyses of the hundreds of unassayed variants should be instructive in future studies.

## Discussion

RT enzymatic activity associated with neurons in the aged human normal and AD cerebral cortex and its relationship to monocistronic L1 ORF2 have not been previously reported. RT activity has been inferred based on L1 retrotransposition, which is postulated to generate neuronal diversity as one of the many forms of SGM (Rehen et al., 2001; Muotri et al., 2005; Singer et al., 2010; Faulkner and Garcia-Perez, 2017; Costantino et al., 2021; Gorbunova et al., 2021). It is largely assumed that L1 retrotransposition utilizes a full-length L1 mRNA sequence to produce insertional mutagenesis, as has been demonstrated in proliferating NPCs via engineered L1 reporter constructs (Muotri et al., 2005; Garcia-Perez et al., 2007; Coufal et al., 2009, 2011). Bulk and single-cell short–read sequencing detection of novel L1 genomic DNA insertion sites in adult human brain cell genomes support somatic retrotransposition of L1 in the human brain (Baillie et al., 2011; Evrony et al., 2012). Prior functional studies have focused on models employing engineered, full-length L1 to study L1 retrotransposition, predominantly utilizing in vitro experimentation on proliferative NPCs, without directly assessing human neurons in vivo or with increasing age (Ostertag et al., 2000,; Muotri et al., 2005; Garcia-Perez et al., 2007; Coufal et al., 2009, 2011; Macia et al., 2017).

To address the question of endogenous brain RT activity and its relationship to L1 mRNA diversity in the human brain, we utilized a multifactorial approach of enzymatic assays, spatial transcriptomics, and PacBio long-read sequencing. Thirty-one human brain PFC and MTG samples with high RNA integrity scores were identified, matched for AD or ND case controls, sex, age, and neuroanatomical location. A wide range (∼5,000×) of RT activity was detected in a vast majority of the brain samples examined, including both ND and AD brains. Neurons were a primary, albeit not only, cell type associated with RT activity, based on neuroanatomical assessments, with the gray matter showing statistically significant increases in RT activity over the white matter. No statistically significant differences in RT activity were identified between ND and AD brains nor different regions of the brain, which may be due to insufficient sampling. However, the mean activity values for AD trended lower than those for ND (Fig. 4), which might reflect the neuronal loss that is a hallmark of terminal neurodegenerative AD. These data support neurons as a major contributor of endogenous RT activity in the human cerebral cortex.

Three classes of endogenous RT genes were initially considered: HERV-pol (HERVk), H-TERT, and full-length L1 (Cong et al., 2002; Baldwin et al., 2022). H-TERT activity was indetectable by FPERT in validation experiments, while subsequent pulldowns for HERV-pol sequences proved negative (only inactive sequences were found), leaving full-length L1 as an expected RT source (Fig. 4*F*,*G*). hTERT and HERVs likely have other functions in the cortex but were not assessed here. Previous spatial analyses of L1 protein expression have been hampered by a lack of reliable ORF2 antibodies, therefore limiting examination to ORF1 (Sur et al., 2017; Bonnifet et al., 2023). We identified clear regional, cellular, and subcellular differences in ORF1 versus ORF2 mRNA expression via L1 ORF1- and ORF2-specific RNAscope probe sets (Fig. 1). Significant neuronal (MAP2+) ORF2 and ORF1 expression was detected by spatial transcriptomics (Fig. 1) via quantitative analysis by H-score, as well as qualitatively through the paucity of overlapping ORF1/ORF2 signal as would be expected for bicistronic L1 ORF1 and ORF2 expression. RT activity correlated with MAP2+ cells’ ORF2 signal but not ORF1 (Fig. 4*D*,*E*), supporting neuronal monocistronic ORF2 expression as a source of RT activity.

Quantitative PCR and short-read sequencing cannot easily detect complete or heterogeneous variant L1 sequences—a majority of short-read sequencing analyses bioinformatically discard nonmapped or multimapping reads, which is especially prevalent with younger and more active L1 variants (Deininger et al., 2016; Shpyleva et al., 2018; Stow et al., 2022; Garza et al., 2023; Rybacki et al., 2023). Utilizing PacBio long-read sequencing combined with L1 enrichment through Twist pulldown probes, we generated the most in-depth long–read human brain L1 transcriptome dataset from multiple brain regions, enabling the capture of mRNAs expressed from polymorphic, somatic, and germline L1s. Long-read sequencing revealed marked diversity of L1 mRNAs that were not predicted from prior short-read sequencing, with significantly higher diversity and expression of monocistronic ORF1 and monocistronic ORF2 transcripts compared with the expected bicistronic L1 transcripts. Approximately 80% of all L1 transcripts were noncoding, and almost no full-length coding L1 (0.01% of L1 reads from only 12 independent variants) was detected, with most individual samples lacking even one full-length L1 (Fig. 3*H*).

A striking feature of the neural L1 transcriptome was its sequence diversity. Pervasive intersample and interindividual uniqueness was evident among the >550 different protein-coding polyadenylated monocistronic ORF2 mRNA sequences (Fig. 3). Interindividual L1 polymorphisms leave open the possibility that diversity arises from the expression of germline L1s unique to the individual, given that the >550 distinct variants exceed the number detected in the hg38 human reference genome by >2× (Table 1). Posttranscriptional mRNA processing and premature polyadenylation of L1 mRNA might also contribute to the increased prevalence of ORF2-only and ORF1-only transcripts, with some prior data suggesting that intact ORF2 transcripts may be a brain-specific phenomenon (Belancio et al., 2010; Rybacki et al., 2023). A not mutually exclusive alternative source is from L1 somatic retroinsertions. Speculatively, the 500,000+ germline L1 sequences could serve as a template and reservoir for the initial generation of novel L1 transcripts including monocistronic ORF2s that themselves could be mutagenized by RT and then somatically retroinserted back into the genome, similar to processes proposed for gencDNAs (Faulkner and Garcia-Perez, 2017; Lee et al., 2018; Kaeser and Chun, 2020). Future advances in single-cell long–read sequencing, combined with functional studies, will help to resolve these important questions about where and how this extreme L1 transcriptomic diversity arises and its likely multiple roles.

Further technical challenges persist. The FPERT assay employs a bacteriophage RNA template which is not specific to L1 ORF2 RT, thus complicating our capacity to correlate endogenous RT activity directly with L1 ORF2. Further research examining direct evidence of the diversity of endogenous ORF1 and ORF2 protein expression is needed. Previous studies have depended on tagging L1 ORF2 constructs for protein detection (which is not applicable within the context of examining endogenous L1 ORF2 variant expression), while other protein analysis techniques rely on reliable and robust antibodies (of which there are currently none for ORF2). Targeted enrichment has the possibility to skew read distribution; however, untargeted long-read sequencing decreases the feasibility of approaching these questions because of the relatively low prevalence of L1 transcription, significant costs associated with long-read sequencing, and difficulty of obtaining large, high-quality human brain samples. Further technological advances will improve the detection and study of ORF2 variants within the human brain.

This picture of L1 transcription within the aging human brain and its neurons differs from the classical view of L1 in multiple ways: (1) full-length bicistronic transcripts required for classical retrotransposition are virtually absent (0.01%); (2) monocistronic ORF1 and ORF2 expression support predominantly independent functions beyond L1 retrotransposition; and (3) highly variable endogenous brain RT activity supports brain-to-brain and within-brain neuroanatomical differences, consistent with the expression of monocistronic, protein-coding ORF2 variants (but not ORF1) that independently tracked with neuronal expression. Examination of even a small subset of the expressed ORF2 variants using in vitro overexpression cell culture assays demonstrated that variants are capable of a vast range of RT activities (0–50× over control transfections) while simultaneously maintaining relatively constant EN activity indicative of coding potential from the same variant species.

Neuronal RT activity mediated by monocistronic ORF2 may promote SGM by RNA retroinsertion of ALUs, SINEs, other transposable elements, and cellular genes via SGR to affect coding and noncoding neuronal genomes and alter DNA content as reported for normal, aging, and AD neurons (Wei et al., 2001; Dewannieux et al., 2003; Westra et al., 2010; Bushman et al., 2015; Lee et al., 2018 , 2020; Park et al., 2019; Mitsunaga et al., 2023). The ND brain may benefit from RT-mediated genome plasticity for improved neuronal function, reflecting a form of cellular memory (Bachiller et al., 2017; Lee et al., 2018; Kaeser and Chun, 2020). Possible disease implications include proinflammatory ssDNA production, activation of the cGAS-STING pathway, creation of DSBs, and senescence, all of which could promote neurodegeneration and other disease endpoints (Feng et al., 1996; De Cecco et al., 2019; Della Valle et al., 2022; Mathavarajah and Dellaire, 2024).

Quantitative and qualitative changes in RT activity mediated by diverse ORF2 variants as identified here could also underlie altered effects of approved and in-development medicines reported to inhibit RT activity, thus explaining contradictory reports on efficacy including the effectiveness of allosteric non-nucleoside RT inhibitors like Efavirenz, developed against the HIV RT heterodimer (Sciamanna et al., 2005; Braz et al., 2010; Dai et al., 2011; Patnala et al., 2013; Banuelos-Sanchez et al., 2019; Baldwin et al., 2024). Recent post hoc real-world analyses of RT inhibitor exposure in HIV+ individuals at risk for AD support beneficial effects in reducing AD incidence (Chow et al., 2024), as does a recent Phase 2a clinical trial (Sullivan et al., 2025). Other suggestive results from ongoing clinical trials are emerging for multiple other neurodegenerative diseases (NCT04993768, NCT04993755, NCT04500847, NCT04552795), supporting a generalizable strategy of inhibiting RT activity produced by translation of specific ORF2 variants in neurons of the aging brain for the prevention and/or treatment of AD and other human brain diseases.

## References

[B1] Altschul S (1997) Gapped BLAST and PSI-BLAST: a new generation of protein database search programs. Nucleic Acids Res 25:3389–3402. 10.1093/nar/25.17.3389 9254694 PMC146917

[B2] Anda DCF, et al. (2016) Cortical neurons gradually attain a post-mitotic state. Cell Res 26:1033–1047. 10.1038/cr.2016.76 27325298 PMC5034108

[B3] Athanikar JN, Badge RM, Moran JV (2004) A YY1-binding site is required for accurate human LINE-1 transcription initiation. Nucleic Acids Res 32:3846–3855. 10.1093/nar/gkh698 15272086 PMC506791

[B4] Bachiller S, del-Pozo-Martín Y, Carrión ÁM (2017) L1 retrotransposition alters the hippocampal genomic landscape enabling memory formation. Brain Behav Immun 64:65–70. 10.1016/j.bbi.2016.12.018 28012829

[B5] Bae T, et al. (2022) Analysis of somatic mutations in 131 human brains reveals ageing-associated hypermutability. Science 377:511–517. 10.1126/science.abm6222 35901164 PMC9420557

[B6] Baillie JK, et al. (2011) Somatic retrotransposition alters the genetic landscape of the human brain. Nature 479:534–537. 10.1038/nature10531 22037309 PMC3224101

[B7] Baldwin ET, et al. (2022) Human endogenous retrovirus-K (HERV-K) reverse transcriptase (RT) structure and biochemistry reveals remarkable similarities to HIV-1 RT and opportunities for HERV-K–specific inhibition. Proc Natl Acad Sci U S A 119:e2200260119. 10.1073/pnas.2200260119 35771941 PMC9271190

[B8] Baldwin ET, et al. (2024) Structures, functions and adaptations of the human LINE-1 ORF2 protein. Nature 626:194–206. 10.1038/s41586-023-06947-z 38096902 PMC10830420

[B9] Bankhead P, et al. (2017) Qupath: Open source software for digital pathology image analysis. Sci Rep 7:16878. 10.1038/s41598-017-17204-5 29203879 PMC5715110

[B10] Banuelos-Sanchez G, et al. (2019) Synthesis and characterization of specific reverse transcriptase inhibitors for mammalian LINE-1 retrotransposons. Cell Chem Biol 26:1095–1109.e14. 10.1016/j.chembiol.2019.04.010 31155508

[B11] Bateman A, et al. (2023) UniProt: the universal protein knowledgebase in 2023. Nucleic Acids Res 51:D523–D531. 10.1093/nar/gkac1052 36408920 PMC9825514

[B12] Belancio VP, Hedges DJ, Deininger P (2006) LINE-1 RNA splicing and influences on mammalian gene expression. Nucleic Acids Res 34:1512–1521. 10.1093/nar/gkl027 16554555 PMC1415225

[B13] Belancio VP, Roy-Engel AM, Pochampally RR, Deininger P (2010) Somatic expression of LINE-1 elements in human tissues. Nucleic Acids Res 38:3909–3922. 10.1093/nar/gkq132 20215437 PMC2896524

[B14] Blankenberg D, et al. (2007) A framework for collaborative analysis of ENCODE data: making large-scale analyses biologist-friendly. Genome Res 17:960–964. 10.1101/gr.5578007 17568012 PMC1891355

[B15] Boissinot S, Sookdeo A (2016) The evolution of line-1 in vertebrates. Genome Biol Evol 8:3485–3507. 10.1093/gbe/evw247 28175298 PMC5381506

[B16] Bonnifet T, Sinnassamy S, Beaudoin OM, Mailly P, Monnet H, Loew D, Lombard B, Servant N, Joshi R, Fuchs J (2023) Steady-state neuron-predominant LINE-1 encoded ORF1p protein and LINE-1 RNA increase with aging in the mouse and human brain. Elife 13:RP100687. 10.7554/eLife.100687.1 PMC12463392 40996791

[B17] Braz VA, Holladay LA, Barkley MD (2010) Efavirenz binding to HIV-1 reverse transcriptase monomers and dimers. Biochemistry 49:601–610. 10.1021/bi901579y 20039714 PMC2896556

[B18] Brouha B, Schustak J, Badge RM, Lutz-Prigge S, Farley AH, Moran JV, Kazazian HH Jr (2003) Hot L1s account for the bulk of retrotransposition in the human population. Proc Natl Acad Sci U S A 100:5280–5285. 10.1073/pnas.0831042100 12682288 PMC154336

[B19] Bushman DM, Kaeser GE, Siddoway B, Westra JW, Rivera RR, Rehen SK, Yung YC, Chun J (2015) Genomic mosaicism with increased amyloid precursor protein (APP) gene copy number in single neurons from sporadic Alzheimer's disease brains. Elife 4:e05116. 10.7554/eLife.05116 25650802 PMC4337608

[B20] Charnaud S, et al. (2022) Pacbio long-read amplicon sequencing enables scalable high-resolution population allele typing of the complex CYP2D6 locus. Commun Biol 5:168. 10.1038/s42003-022-03102-8 35217695 PMC8881578

[B21] Chow TW, Raupp M, Reynolds MW, Li S, Kaeser GE, Chun J (2024) Nucleoside reverse transcriptase inhibitor exposure Is associated with lower Alzheimer's disease risk: a retrospective cohort proof-of-concept study. Pharmaceuticals 17:408. 10.3390/ph17040408 38675371 PMC11053431

[B22] Cock PJ, Chilton JM, Grüning B, Johnson JE, Soranzo N (2015) NCBI BLAST+ integrated into Galaxy. Gigascience 4:39. 10.1186/s13742-015-0080-7 26336600 PMC4557756

[B23] Cong YS, Wright WE, Shay JW (2002) Human telomerase and its regulation. Microbiol Mol Biol Rev 66:407–425. 10.1128/MMBR.66.3.407-425.2002 12208997 PMC120798

[B24] Costantino I, Nicodemus J, Chun J (2021) Genomic mosaicism formed by somatic variation in the aging and diseased brain. Genes 12:1071. 10.3390/genes12071071 34356087 PMC8305509

[B25] Coufal NG, Garcia-Perez JL, Peng GE, Yeo GW, Mu Y, Lovci MT, Morell M, O'Shea KS, Moran JV, Gage FH (2009) L1 retrotransposition in human neural progenitor cells. Nature 460:1127–1131. 10.1038/nature08248 19657334 PMC2909034

[B26] Coufal NG, Garcia-Perez JL, Peng GE, Marchetto MC, Muotri AR, Mu Y, Carson CT, Macia A, Moran JV, Gage FH (2011) Ataxia telangiectasia mutated (ATM) modulates long interspersed element-1 (L1) retrotransposition in human neural stem cells. Proc Natl Acad Sci U S A 108:20382–20387. 10.1073/pnas.1100273108 22159035 PMC3251057

[B27] Dai L, Huang Q, Boeke JD (2011) Effect of reverse transcriptase inhibitors on LINE-1 and Ty1 reverse transcriptase activities and on LINE-1 retrotransposition. BMC Biochem 12:18. 10.1186/1471-2091-12-18 21545744 PMC3103432

[B28] De Cecco M, et al. (2019) L1 drives IFN in senescent cells and promotes age-associated inflammation. Nature 566:73–78. 10.1038/s41586-018-0784-9 30728521 PMC6519963

[B29] Deininger P, et al. (2016) A comprehensive approach to expression of L1 loci. Nucleic Acids Res 45:e31. 10.1093/nar/gkw1067 27899577 PMC5389711

[B30] Della Valle F, et al. (2022) LINE-1 RNA causes heterochromatin erosion and is a target for amelioration of senescent phenotypes in progeroid syndromes. Sci Transl Med 14:eabl6057. 10.1126/scitranslmed.abl6057 35947677

[B31] Dewannieux M, Esnault C, Heidmann T (2003) LINE-mediated retrotransposition of marked Alu sequences. Nat Genet 35:41–48. 10.1038/ng1223 12897783

[B32] Dombroski BA, Mathias SL, Nanthakumar E, Scott AF, Kazazian HH Jr (1991) Isolation of an active human transposable element. Science 254:1805–1808. 10.1126/science.1662412 1662412

[B33] Erwin JA, Marchetto MC, Gage FH (2014) Mobile DNA elements in the generation of diversity and complexity in the brain. Nat Rev Neurosci 15:497–506. 10.1038/nrn3730 25005482 PMC4443810

[B34] Evrony GD, et al. (2012) Single-neuron sequencing analysis of L1 retrotransposition and somatic mutation in the human brain. Cell 151:483–496. 10.1016/j.cell.2012.09.035 23101622 PMC3567441

[B35] Faulkner GJ, Garcia-Perez JL (2017) L1 mosaicism in mammals: extent, effects, and evolution. Trends Genet 33:802–816. 10.1016/j.tig.2017.07.004 28797643

[B36] Feng Q, Moran JV, Kazazian HH, Boeke JD Jr (1996) Human L1 retrotransposon encodes a conserved endonuclease required for retrotransposition. Cell 87:905–916. 10.1016/S0092-8674(00)81997-2 8945517

[B37] Garcia-Perez JL, Doucet AJ, Bucheton A, Moran JV, Gilbert N (2007) Distinct mechanisms for trans-mediated mobilization of cellular RNAs by the LINE-1 reverse transcriptase. Genome Res 17:602–611. 10.1101/gr.5870107 17416749 PMC1855177

[B38] Garza R, et al. (2023) LINE-1 retrotransposons drive human neuronal transcriptome complexity and functional diversification. Sci Adv 9:eadh9543. 10.1126/sciadv.adh9543 37910626 PMC10619931

[B39] Gilbert N, Lutz S, Morrish TA, Moran JV (2005) Multiple fates of L1 retrotransposition intermediates in cultured human cells. Mol Cell Biol 25:7780–7795. 10.1128/MCB.25.17.7780-7795.2005 16107723 PMC1190285

[B40] Gorbunova V, et al. (2021) The role of retrotransposable elements in ageing and age-associated diseases. Nature 596:43–53. 10.1038/s41586-021-03542-y 34349292 PMC8600649

[B41] Guo C, Jeong H-H, Hsieh Y-C, Klein H-U, Bennett DA, De Jager PL, Liu Z, Shulman JM (2018) Tau activates transposable elements in Alzheimer’s disease. Cell Rep 23:2874–2880. 10.1016/j.celrep.2018.05.004 29874575 PMC6181645

[B42] Jolly S, Lang V, Koelzer VH, Sala Frigerio C, Magno L, Salinas PC, Whiting P, Palomer E (2019) Single-cell quantification of mRNA expression in the human brain. Sci Rep 9:12353. 10.1038/s41598-019-48787-w 31451701 PMC6710275

[B43] Kaer K, Branovets J, Hallikma A, Nigumann P, Speek M (2011) Intronic L1 retrotransposons and nested genes cause transcriptional interference by inducing intron retention, exonization and cryptic polyadenylation. PLoS One 6:e26099. 10.1371/journal.pone.0026099 22022525 PMC3192792

[B44] Kaeser G, Chun J (2020) Brain cell somatic gene recombination and its phylogenetic foundations. J Biol Chem 295:12786–12795. 10.1074/jbc.REV120.009192 32699111 PMC7476723

[B45] Kaul T, Morales ME, Sartor AO, Belancio VP, Deininger P (2020) Comparative analysis on the expression of L1 loci using various RNA-seq preparations. Mob DNA 11:2. 10.1186/s13100-019-0194-z 31921361 PMC6945437

[B46] Kinner A, Wu W, Staudt C, Iliakis G (2008) Gamma-H2AX in recognition and signaling of DNA double-strand breaks in the context of chromatin. Nucleic Acids Res 36:5678–5694. 10.1093/nar/gkn550 18772227 PMC2553572

[B47] Kohany O, Gentles AJ, Hankus L, Jurka J (2006) Annotation, submission and screening of repetitive elements in Repbase: RepbaseSubmitter and Censor. BMC Bioinformatics 7:474. 10.1186/1471-2105-7-474 17064419 PMC1634758

[B49] Lanciano S, Cristofari G (2020) Measuring and interpreting transposable element expression. Nat Rev Genet 21:721–736. 10.1038/s41576-020-0251-y 32576954

[B50] Lander E, Linton L, Birren B, Nusbaum C, Zody M, Baldwin J (2001) Initial sequencing and analysis of the human genome. Nature 409:860–921. 10.1038/35057062 11237011

[B51] Lee MH, et al. (2018) Somatic APP gene recombination in Alzheimer’s disease and normal neurons. Nature 563:639–645. 10.1038/s41586-018-0718-6 30464338 PMC6391999

[B52] Lee MH, Liu CS, Zhu Y, Kaeser GE, Rivera R, Romanow WJ, Kihara Y, Chun J (2020) Reply to: APP gene copy number changes reflect exogenous contamination. Nature 584:E29–E33. 10.1038/s41586-020-2523-2 32814882 PMC8522531

[B53] Lee YN, Bieniasz PD (2007) Reconstitution of an infectious human endogenous retrovirus. PLoS Pathog 3:e10. 10.1371/journal.ppat.0030010 17257061 PMC1781480

[B54] Leung SK, et al. (2021) Full-length transcript sequencing of human and mouse cerebral cortex identifies widespread isoform diversity and alternative splicing. Cell Rep 37:110022. 10.1016/j.celrep.2021.110022 34788620 PMC8609283

[B55] Logsdon GA, Vollger MR, Eichler EE (2020) Long-read human genome sequencing and its applications. Nat Rev Genet 21:597–614. 10.1038/s41576-020-0236-x 32504078 PMC7877196

[B56] Ma YK, Khan AS (2009) Evaluation of different RT enzyme standards for quantitation of retroviruses using the single-tube fluorescent product-enhanced reverse transcriptase assay. J Virol Methods 157:133–140. 10.1016/j.jviromet.2009.01.002 19186191

[B57] Macciardi F, et al. (2022) A retrotransposon storm marks clinical phenoconversion to late-onset Alzheimer’s disease. GeroScience 44:1525–1550. 10.1007/s11357-022-00580-w 35585302 PMC9213607

[B58] Macia A, et al. (2017) Engineered LINE-1 retrotransposition in nondividing human neurons. Genome Res 27:335–348. 10.1101/gr.206805.116 27965292 PMC5340962

[B59] Mathavarajah S, Dellaire G (2024) LINE-1: an emerging initiator of cGAS-STING signalling and inflammation that is dysregulated in disease. Biochem Cell Biol 102:38–46. 10.1139/bcb-2023-0134 37643478

[B60] Mathias SL, Scott AF, Kazazian HH Jr, Boeke JD, Gabriel A (1991) Reverse transcriptase encoded by a human transposable element. Science 254:1808–1810. 10.1126/science.1722352 1722352

[B61] Metsky HC, et al. (2019) Capturing sequence diversity in metagenomes with comprehensive and scalable probe design. Nat Biotechnol 37:160–168. 10.1038/s41587-018-0006-x 30718881 PMC6587591

[B62] Mitsunaga S, Fujito N, Nakaoka H, Imazeki R, Nagata E, Inoue I (2023) Detection of APP gene recombinant in human blood plasma. Sci Rep 13:21703. 10.1038/s41598-023-48993-7 38066066 PMC10709617

[B63] Moran JV, Holmes SE, Naas TP, DeBerardinis RJ, Boeke JD, Kazazian HH Jr (1996) High frequency retrotransposition in cultured mammalian cells. Cell 87:917–927. 10.1016/S0092-8674(00)81998-4 8945518

[B64] Muotri AR, Chu VT, Marchetto MCN, Deng W, Moran JV, Gage FH (2005) Somatic mosaicism in neuronal precursor cells mediated by L1 retrotransposition. Nature 435:903–910. 10.1038/nature03663 15959507

[B65] Naufer MN, Furano AV, Williams MC (2019) Protein-nucleic acid interactions of LINE-1 ORF1p. Semin Cell Dev Biol 86:140–149. 10.1016/j.semcdb.2018.03.019 29596909 PMC6428221

[B66] Ostertag EM, Prak ET, DeBerardinis RJ, Moran JV, Kazazian HH Jr (2000) Determination of L1 retrotransposition kinetics in cultured cells. Nucleic Acids Res 28:1418–1423. 10.1093/nar/28.6.1418 10684937 PMC111040

[B67] Pabis K, Barardo D, Selvarajoo K, Gruber J, Kennedy BK (2023) A concerted increase in readthrough and intron retention drives transposon expression during aging and senescence. Elife 12:RP87811. 10.7554/eLife.87811.3 PMC10990488 38567944

[B68] Park JS, et al. (2019) Brain somatic mutations observed in Alzheimer’s disease associated with aging and dysregulation of tau phosphorylation. Nat Commun 10:3090. 10.1038/s41467-019-11000-7 31300647 PMC6626023

[B69] Pascarella G, et al. (2022) Recombination of repeat elements generates somatic complexity in human genomes. Cell 185:3025–3040.e6. 10.1016/j.cell.2022.06.032 35882231

[B70] Patnala R, Lee S-H, Dahlstrom JE, Ohms S, Chen L, Dheen ST, Rangasamy D (2013) Inhibition of LINE-1 retrotransposon-encoded reverse transcriptase modulates the expression of cell differentiation genes in breast cancer cells. Breast Cancer Res Treat 143:239–253. 10.1007/s10549-013-2812-7 24337508 PMC3889873

[B71] Penzkofer T, Jäger M, Figlerowicz M, Badge R, Mundlos S, Robinson PN, Zemojtel T (2017) L1base 2: more retrotransposition-active LINE-1s, more mammalian genomes. Nucleic Acids Res 45:D68–D73. 10.1093/nar/gkw925 27924012 PMC5210629

[B72] Pfaff AL, Bubb VJ, Quinn JP, Koks S (2020) An increased burden of highly active retrotransposition competent L1s Is associated with Parkinson’s disease risk and progression in the PPMI cohort. Int J Mol Sci 21:6562. 10.3390/ijms21186562 32911699 PMC7554759

[B73] Piovesan A, Antonaros F, Vitale L, Strippoli P, Pelleri MC, Caracausi M (2019) Human protein-coding genes and gene feature statistics in 2019. BMC Res Notes 12:315. 10.1186/s13104-019-4343-8 31164174 PMC6549324

[B74] Quinlan AR, Hall IM (2010) BEDTools: a flexible suite of utilities for comparing genomic features. Bioinformatics 26:841–842. 10.1093/bioinformatics/btq033 20110278 PMC2832824

[B75] Rangwala SH, Zhang L, Kazazian HH Jr (2009) Many LINE1 elements contribute to the transcriptome of human somatic cells. Genome Biol 10:R100. 10.1186/gb-2009-10-9-r100 19772661 PMC2768975

[B76] Rehen SK, McConnell MJ, Kaushal D, Kingsbury MA, Yang AH, Chun J (2001) Chromosomal variation in neurons of the developing and adult mammalian nervous system. Proc Natl Acad Sci U S A 98:13361–13366. 10.1073/pnas.231487398 11698687 PMC60876

[B77] Rice P, Longden I, Bleasby A (2000) EMBOSS: the European Molecular Biology Open Software Suite. Trends Genet 16:276–277. 10.1016/S0168-9525(00)02024-2 10827456

[B78] Richardson SR, Morell S, Faulkner GJ (2014) L1 retrotransposons and somatic mosaicism in the brain. Annu Rev Genet 48:1–27. 10.1146/annurev-genet-120213-092412 25036377

[B79] Rohrback S, Siddoway B, Liu CS, Chun J (2018) Genomic mosaicism in the developing and adult brain. Dev Neurobiol 78:1026–1048. 10.1002/dneu.22626 30027562 PMC6214721

[B80] Rouchka E, Montoya-Durango DE, Stribinskis V, Ramos K, Kalbfleisch T (2010) Assessment of genetic variation for the LINE-1 retrotransposon from next generation sequence data. BMC Bioinformatics 11:S12. 10.1186/1471-2105-11-S9-S12 21044359 PMC2967742

[B81] Rybacki K, Xia M, Ahsan MU, Xing J, Wang K (2023) Assessing the expression of Long INterspersed Elements (LINEs) via long-read sequencing in diverse human tissues and cell lines. Genes (Basel) 14:1893. 10.3390/genes14101893 37895242 PMC10606529

[B82] Sciamanna I, et al. (2005) Inhibition of endogenous reverse transcriptase antagonizes human tumor growth. Oncogene 24:3923–3931. 10.1038/sj.onc.1208562 15806170

[B83] Shpyleva S, Melnyk S, Pavliv O, Pogribny I, Jill James S (2018) Overexpression of LINE-1 retrotransposons in autism brain. Mol Neurobiol 55:1740–1749. 10.1007/s12035-017-0421-x 28220356

[B84] Singer T, McConnell MJ, Marchetto MCN, Coufal NG, Gage FH (2010) LINE-1 retrotransposons: mediators of somatic variation in neuronal genomes? Trends Neurosci 33:345–354. 10.1016/j.tins.2010.04.001 20471112 PMC2916067

[B85] Stow EC, Baddoo M, LaRosa AJ, LaCoste D, Deininger P, Belancio V (2022) SCIFER: approach for analysis of LINE-1 mRNA expression in single cells at a single locus resolution. Mob DNA 13:21. 10.1186/s13100-022-00276-0 36028901 PMC9413895

[B86] Sullivan AC, et al. (2025) A phase IIa clinical trial to evaluate the effects of anti-retroviral therapy in Alzheimer's disease (ART-AD). NPJ Dement 1:2. 10.1038/s44400-024-00001-z 40104524 PMC11917871

[B87] Sur D, Kustwar RK, Budania S, Mahadevan A, Hancks DC, Yadav V, Shankar SK, Mandal PK (2017) Detection of the LINE-1 retrotransposon RNA-binding protein ORF1p in different anatomical regions of the human brain. Mob DNA 8:17. 10.1186/s13100-017-0101-4 29201157 PMC5700708

[B88] Swergold GD (1990) Identification, characterization, and cell specificity of a human LINE-1 promoter. Mol Cell Biol 10:6718–6729. 10.1128/mcb.10.12.6718-6729.1990 1701022 PMC362950

[B89] Tardaguila M, et al. (2018) SQANTI: extensive characterization of long-read transcript sequences for quality control in full-length transcriptome identification and quantification. Genome Res 28:396–411. 10.1101/gr.222976.117 29440222 PMC5848618

[B90] Thawani A, Ariza AJF, Nogales E, Collins K (2024) Template and target site recognition by human LINE-1 in retrotransposition. Nature 626:186–193. 10.1038/s41586-023-06933-5 38096901 PMC10830416

[B91] The Galaxy Community (2022) The Galaxy platform for accessible, reproducible and collaborative biomedical analyses: 2022 update. Nucleic Acids Res 50:W345–W351. 10.1093/nar/gkac247 35446428 PMC9252830

[B92] Upton KR, et al. (2015) Ubiquitous L1 mosaicism in hippocampal neurons. Cell 161:228–239. 10.1016/j.cell.2015.03.026 25860606 PMC4398972

[B93] von Bartheld CS, Bahney J, Herculano-Houzel S (2016) The search for true numbers of neurons and glial cells in the human brain: a review of 150 years of cell counting. Journal of Comparative Neurology 524:3865–3895. 10.1002/cne.24040 27187682 PMC5063692

[B94] Wagstaff BJ, Barnerssoi M, Roy-Engel AM (2011) Evolutionary conservation of the functional modularity of primate and murine LINE-1 elements. PLoS One 6:e19672. 10.1371/journal.pone.0019672 21572950 PMC3091869

[B95] Wei W, Gilbert N, Ooi SL, Lawler JF, Ostertag EM, Kazazian HH, Boeke JD, Moran JV (2001) Human L1 retrotransposition: cisPreference versus trans complementation. Mol Cell Biol 21:1429–1439. 10.1128/MCB.21.4.1429-1439.2001 11158327 PMC99594

[B96] Wei W, et al. (2019) Frequency and signature of somatic variants in 1461 human brain exomes. Genet Med 21:904–912. 10.1038/s41436-018-0274-3 30214067 PMC6544539

[B97] Wenger AM, et al. (2019) Accurate circular consensus long-read sequencing improves variant detection and assembly of a human genome. Nat Biotechnol 37:1155–1162. 10.1038/s41587-019-0217-9 31406327 PMC6776680

[B98] Westra JW, Rivera RR, Bushman DM, Yung YC, Peterson SE, Barral S, Chun J (2010) Neuronal DNA content variation (DCV) with regional and individual differences in the human brain. J Comp Neurol 518:3981–4000. 10.1002/cne.22436 20737596 PMC2932632

